# Experimental identification of preQ_1_-binding RNAs in the pathogenic bacterium *Listeria monocytogenes*

**DOI:** 10.1039/d5cb00102a

**Published:** 2025-10-14

**Authors:** Malou Hanisch, Laurin Flemmich, Christoph Mitteregger, Ingo Bauer, Cristian A. Velandia-Huerto, Ivo Hofacker, Ronald Micura, Alexandra Lusser

**Affiliations:** a Institute of Molecular Biology, Biocenter, Medical University of Innsbruck Innrain 80-82 6020 Innsbruck Austria alexandra.lusser@i-med.ac.at; b Institute of Organic Chemistry, Center for Molecular Biosciences Innsbruck, University of Innsbruck Innrain 80-82 6020 Innsbruck Austria ronald.micura@uibk.ac.at; c Department of Theoretical Chemistry, University of Vienna Währinger Straße 17 1090 Vienna Austria; d Center for Anatomy and Cell Biology, Medical University of Vienna Schwarzspanierstraße 17 1090 Vienna Austria; e Research Group Bioinformatics and Computational Biology, University of Vienna Währinger Straße 29 1090 Vienna Austria

## Abstract

Riboswitches are widespread regulatory RNA modules in bacteria, with many different classes already identified and even more yet to be discovered. Traditionally, the identification of riboswitches has relied on bioinformatic analyses and genetic screens. In this work, we explored the possibility of identifying and characterizing predicted and novel riboswitches using an affinity purification-based approach with a functionalized preQ_1_ ligand. We successfully enriched a predicted preQ_1_ riboswitch from *L. monocytogenes* total RNA. Biophysical characterization revealed that this riboswitch can simultaneously bind two ligand molecules and functions as a regulator of translation *in vivo*. Furthermore, a transcriptome-wide pull-down experiment resulted in strong preQ_1_-dependent enrichment of several candidate sequences. Characterization of the *lmo2684* candidate mRNA revealed a preQ_1_ riboswitch-like sequence in its 5′ untranslated region. Notably, preQ_1_ allowed translation of an upstream open reading frame in this region by promoting stop codon readthrough. Our findings highlight the utility of ligand-based pull-down strategies for enriching mRNAs with aptamers that elude computational detection and may possess undiscovered functions.

## Abstract 2

Pull-down, chemical, and functional analysis of preQ_1_-binding RNAs: Validation of a predicted preQ_1_ riboswitch binding two preQ_1_ molecules and discovery of an mRNA element regulating upstream open reading frame usage in a preQ_1_-dependent manner.

## Graphical abstract

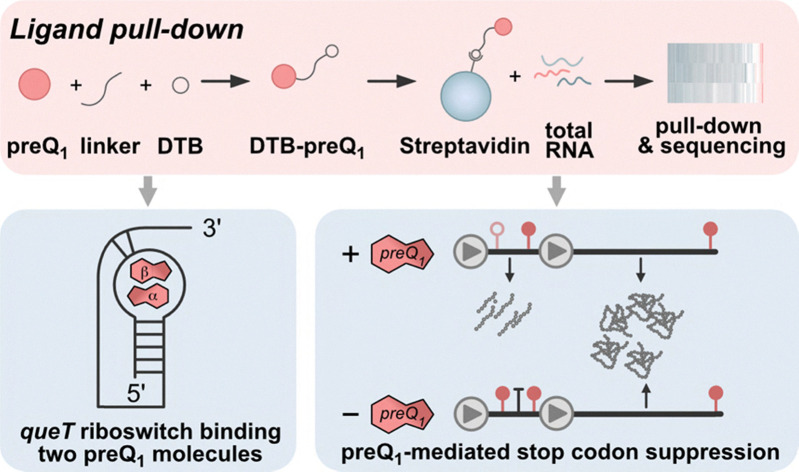

## Introduction

Riboswitches are RNA-based control elements located primarily in the 5′ untranslated regions (5′ UTRs) of bacterial mRNAs. Typically, they bind elemental ions or cellular metabolites with high affinity resulting in the activation or, more commonly, inactivation of transcription or translation of the downstream gene.^1–3^ The aptamer region of the riboswitch folds into secondary and tertiary structures to ensure specific ligand binding. Ligand binding to the aptamer region triggers folding changes involving the so-called expression platform that comprises, for instance, the Shine–Dalgarno sequence or transcription terminator sequences. This can result in sequestration or exposure of these regulatory elements affecting the expression of the associated gene.^4,5^ A well-studied riboswitch binds the 7-aminomethyl-7-deazaguanine molecule prequeuosine 1 (preQ_1_) that is a precursor of the queuosine (Q) purine nucleoside.^6,7^ Q can be found in the wobble position of the anticodons of tRNA^Asn^, tRNA^Asp^, tRNA^His^ and tRNA^Tyr^.^8,9^ It is thought to fine-tune gene regulation by stabilizing anticodon-codon interactions.^10^ In eukaryotes, Q was shown to be involved in cellular differentiation and proliferation,^11–13^ tyrosine biosynthesis^14^ and response to hypoxic stress.^15^ Q-deficient bacteria have growth defects,^16^ diminished virulence^17^ and reduced viability under stress conditions.^18^ Some bacteria can synthesize Q *de novo* while others transport or salvage it from the gut or other microbes.^19^ In bacteria that produce Q *de novo*, the preQ_1_ riboswitch often controls gene expression of the *queCDEF* operon, which encodes several proteins involved in Q-tRNA synthesis.^6^ Many bacteria that rely on the transport or salvage of preQ_1_ were shown to control preQ_1_ transporter genes, such as *yhhQ*, *queT* and *qrtT*, or preQ_1_ salvage genes, such as *queL* and *queK*, by preQ_1_ riboswitches.^20–22^ Because of their small size and physiological impact, they have attracted significant interest as potential antibacterial drug targets.^23^

There are three classes of preQ_1_ riboswitches.^7^ preQ_1_ class I (preQ_1_-I) riboswitches are among the smallest known riboswitches, while preQ_1_ class II and class III (preQ_1_-II, preQ_1_-III) are more complex. However, they all bind preQ_1_ with similar affinities.^7^ In addition, bioinformatic analyses revealed the presence of three subtypes within preQ_1_-I with subtype 1 being the most widespread among different types of bacteria, while types 2 and 3 appear to be less common.^7^ Interestingly, preQ_1_-I type 1 riboswitches have recently been found to enable cooperative binding of two preQ_1_ molecules.^24^

In the past two decades, more than 55 riboswitch classes have been identified,^4,25^ yet the number of undiscovered riboswitches is estimated to be in the range of several hundreds to thousands.^26^ Bioinformatic searches have been extremely useful in discovering novel riboswitch classes by combining sequence data, structure prediction, functional information and comparative genomics,^26^ yet additional experimental methods should be considered as well. For example, a recently developed photocrosslinking-based approach was employed to profile preQ_1_–RNA interactions by affinity enrichment.^27^ Moreover, various approaches utilizing covalent crosslinking combined with affinity enrichment have demonstrated their effectiveness as powerful tools for investigating the interactions between a variety of small molecules and RNA (*e.g.* ref. 28–39).

In this work, we employed a ligand-based approach to identify new preQ_1_-binding RNAs. To this end, we generated a biotinylated version of preQ_1_ to be used for streptavidin pull-down of potential preQ_1_ riboswitch- and/or aptamer-containing mRNAs from the pathogenic bacterium *Listeria monocytogenes*. We demonstrate the successful enrichment and functional characterization of the predicted preQ_1_ riboswitch associated with the *queT* mRNA encoding a preQ_1_ transporter. In addition, we present the application of the pull-down strategy in the identification of novel preQ_1_ targets in the *L. monocytogenes* transcriptome.

## Results and discussion

### Synthesis and *in vitro* characterization of a biotin-preQ_1_ conjugate with high affinity to a known riboswitch

**Fig. 1:**
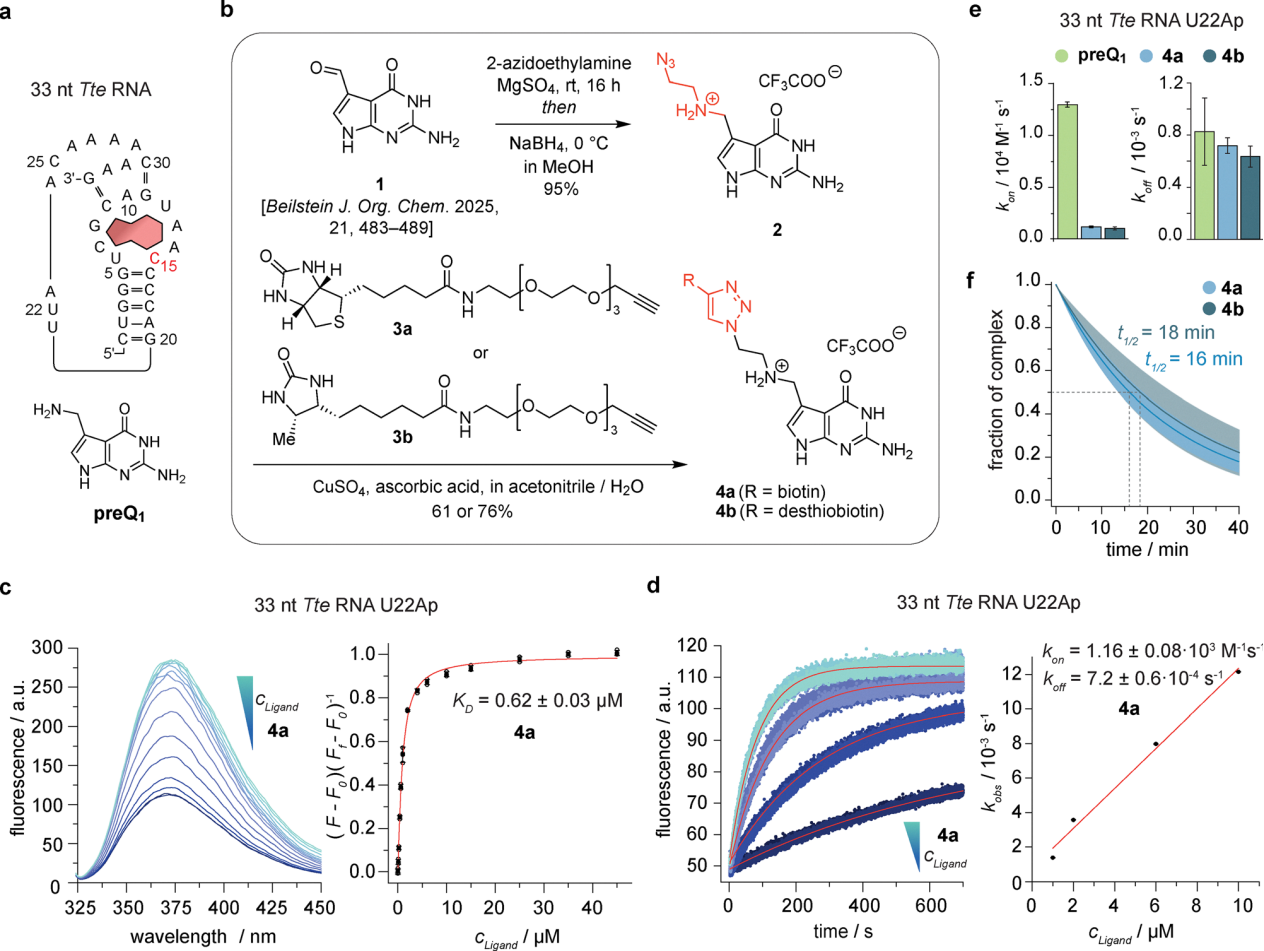
Synthesis and characterization of a preQ_1_-biotin conjugate. (a) Sequence and secondary structure of the preQ_1_ class-I riboswitch from *Thermoanaerobacter tengcongensis* (*Tte*) and chemical structure of its cognate ligand preQ_1_; C15 (red) pairs to preQ_1_ in Watson–Crick mode. (b) Synthesis of preQ_1_-biotin conjugates 4a and 4b. (c) Affinity (*K*_D_) determination of 4a and *Tte* RNA using a 2-aminopurine fluorescence assay. (d) Kinetics (*k*_on_) determination of 4a and *Tte* RNA. (e) Bar graph comparing ligand binding and unbinding kinetics of preQ_1_, 4a and 4b; rate constants are reported as fit value ± fit error that were extracted from the individual conversion *versus* time plots (shown in panel (d) and Fig. S5). (f) Fraction of complex as a function of time simulated for the off-rate of ligands 4a and 4b including the 95% confidence band. All experiments were performed in triplicate, individual data points are depicted as open circles, filled circles correspond to mean values (± s.e.m). Fitted values are provided as mean ± SD.

Starting point of our study was the well-characterized preQ_1_ class I riboswitch of *Thermoanaerobacter tengcongensis* (*Tte*) (Fig. 1a).^40^ Based on existing crystal structures,^40^ we decided to attach a biotin (or desthiobiotin) moiety *via* a short ethylene glycol linker to the 7-aminomethyl group of preQ_1_, because little interference with the actual ligand recognition by the binding pocket is expected. Therefore, reductive amination of compound 1^41^ in the presence of 2-azidoethylamine was conducted first and furnished the azido derivative of preQ_1_ (2) in excellent yields (Fig. 1b and Fig. S1). Then, standard click reaction with commercially available biotin- or desthiobiotin alkyne derivatives 3a and 3b, respectively, gave the desired preQ_1_-biotin and preQ_1_-desthiobiotin (DTB-preQ_1_) conjugates 4a and 4b (Fig. S2–S4). Next, we determined the affinity of 4a to the 33 nt *Tte* RNA aptamer using a previously established fluorescence assay^42^ based on a 2-aminopurine RNA mutant (*Tte* preQ_1_ U22Ap) (Fig. 1c and Fig. S5). The obtained *K*_D_ value of 0.62 μM (4a) was about 10-fold higher compared to the affinity determined for non-functionalized preQ_1_ (*K*_D_(preQ_1_) = 64 nM; Fig. S5). Furthermore, the rate *k*_on_ of complex formation was determined to be 1.16 × 10^3^ M^−1^ s^−1^ (4a) (Fig. 1d), which is 11-fold slower compared to the on-rate obtained for non-functionalized preQ_1_ (*k*_on_(preQ_1_) = 1.3 × 10^4^ M^−1^ s^−1^; Fig. S5). Interestingly the off-rates of the two systems were comparable (*k*_off_ ∼ 7.5 × 10^−4^ s^−1^, Fig. 1d and Fig. S5). Of note, results obtained with 4b were highly similar to those derived from 4a (Fig. S5 and Fig. 1e). Therefore, given the corresponding ligand–RNA complex half-life of approximately 15 min (Fig. 1e and f), pulldown experiments should be feasible.

### Enrichment of *queT* mRNA from total *L. monocytogenes* RNA

**Fig. 2:**
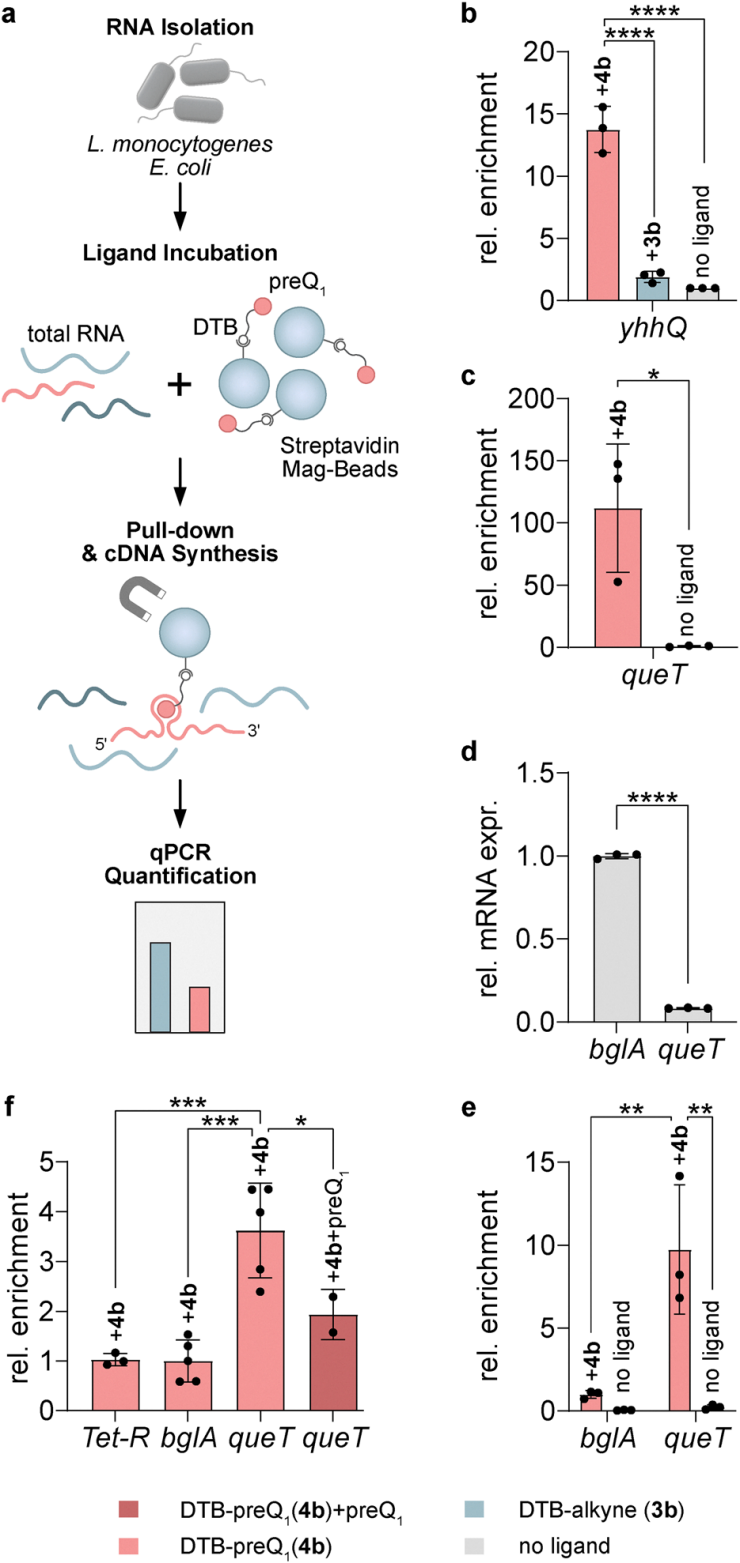
A functionalized preQ_1_ ligand allows for efficient enrichment of cognate riboswitches. (a) Scheme of experimental approach. Total RNA from bacteria was incubated with streptavidin magnetic beads pre-loaded with desthiobiotin (DTB)-coupled preQ_1_ (4b). RNA bound to the beads was analyzed by on-bead cDNA synthesis followed by qPCR (RT-qPCR). (b) and (c) Enrichment of preQ_1_-riboswitch-containing *yhhQ* mRNA from *E. coli* (b) and *queT* mRNA from *L. monocytogenes* (c) compared to pull-down with DTB-alkyne (3b) or no ligand. (d) *queT* mRNA expression levels are shown relative to the housekeeping gene *bglA*. (e) DTB-preQ_1_ (4b) or no-ligand pull-down experiments with *in vitro* transcripts of *queT* and *bglA* mixed in a 1 : 1 ratio. (f) Enrichment of *queT* mRNA compared to spike-in *Tet-R* or endogenous *bglA* mRNA or after addition of excess non-functionalized preQ_1_. All experiments were performed at least three times and mean values ± SD are shown. Significance was calculated using one-way ANOVA ((b) and (e)), unpaired *t*-test ((c) and (d)) and two-way ANOVA (f) (*p < 0.1; **p < 0.01; ***p < 0.001; ****p < 0.0001). In panels (b), (c) and (f) fold-enrichment of the respective mRNA targets relative to pull-down reactions without ligand was calculated. Color coding in panels (b)–(f) is explained in the legend.

To set up an affinity purification strategy for the enrichment of preQ_1_-aptamer containing sequences based on functionalized preQ_1_ compound 4b, we chose the established preQ_1_ riboswitch in the mRNA of *Escherichia coli yhhQ*^43^ as a target (Fig. 2a). Total RNA was isolated from bacteria and incubated with streptavidin magnetic beads that had been preincubated with DTB-preQ_1_ (4b). After extensive washing, bound RNA was reverse transcribed on the beads and qPCR was used to estimate the extent of enrichment. To control for unspecific binding of the RNA to the beads and/or the DTB-linker moiety, control reactions were performed with unmodified preQ_1_, the DTB-linker (DTB-alkyne 3b; Fig. 1b) or no ligand. The results show that *yhhQ* was strongly enriched in pull-down reactions with DTB-preQ_1_ while negligible amounts were precipitated in the negative control reactions (Fig. 2b). Next, we investigated whether we can also enrich mRNAs with bioinformatically predicted but not yet experimentally validated preQ_1_-riboswitch sequences. To this end, we performed a pull-down experiment with total RNA from *L. monocytogenes*. According to predictions, *L. monocytogenes* contains a single mRNA containing a preQ_1_-I riboswitch.^7,23^ That mRNA encodes the preQ_1_/preQ_0_ transporter QueT.^21^ qPCR analysis of the RNA associated with DTB-preQ_1_-streptavidin beads indeed revealed robust enrichment of *queT* mRNA compared to no-ligand pull-down, even though *queT* exhibited low levels of expression under the growth conditions used (Fig. 2c and d). To assess the specificity of the pull-down reaction under controlled conditions, RNA fragments comprising the 5′UTR (65 nt) and first 207 nt of the coding region of *queT* on one hand, and the 5′UTR (34 nt) and first 225 nt of beta glucosidase *bglA* RNA on the other hand were generated by *in vitro* transcription (IVT). The latter is considered a housekeeping gene with moderate expression^44^ that does not contain any predicted preQ_1_ riboswitch-related sequence in its 5′UTR. Pull-down assays with an equimolar mixture of both fragments demonstrated stronger enrichment of *queT* compared to *bglA* (Fig. 2e). Pull-down with total RNA from *L. monocytogenes* that was spiked with *in vitro* transcribed *TetR* mRNA as a negative control revealed similar results. Furthermore, addition of excess free preQ_1_ to the RNA diminished the binding of *queT* mRNA to DTB-preQ_1_-streptavidin beads underscoring binding specificity (Fig. 2f).

Together these results show that a ligand-based pull-down strategy is suitable for the enrichment of mRNAs bearing the cognate aptamer from total bacterial RNA preparations.

### Functional characterization of *L. monocytogenes queT* riboswitch

**Fig. 3:**
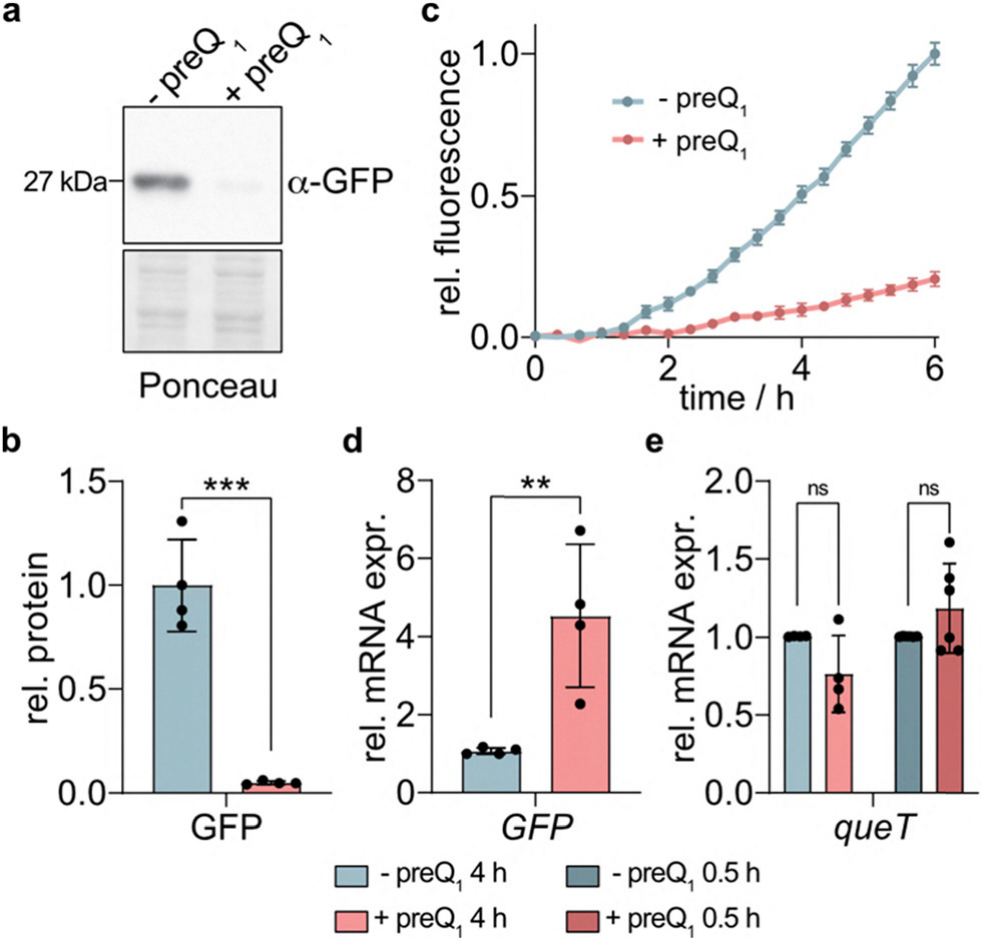
The preQ_1_ riboswitch of *L. monocytogenes queT* regulates protein translation. The 5′UTR region of *L. monocytogenes queT* was cloned into pQE70 expression vector upstream of GFP, and GFP production was tested in *E. coli* strain JW0434. (a) Four hours after addition of 1 mM preQ_1_, cells were harvested, homogenized and subjected to SDS-PAGE and immunoblotting using an α-GFP antibody. (b) Western blot signals of three independent experiments were quantified and normalized against a section of the Ponceau-stained membrane. Mean values ± SD are shown relative to the values obtained from cultures that were handled identically except that no preQ_1_ was added. (c) Direct fluorescence measurements of *E. coli* reporter cells with or without preQ_1_. Data represent mean ± SD from three technical replicates. (d) RT-qPCR analysis of *E. coli* GFP-reporter cells after 6 h incubation with 1 mM preQ_1_. *C*_t_ values for the *GFP* target were normalized against 16S rRNA and are expressed relative to the mock-treated condition (2^−ΔΔ*C*_t_^). Data represent mean ± SD of four independent experiments. (e) RT-qPCR analysis of *L. monocytogenes* cells for *queT* mRNA expression in the absence or presence of 1 mM preQ_1_ for the indicated periods of time. *queT* signals were normalized against *bglA*, and are expressed relative to mock-treated controls. Mean ± SD of 4–6 independent experiments is shown. Statistical significance in panels (b), (d) and (e) was determined by unpaired *t* test and one-way ANOVA (ns, not significant; **p < 0.01; ****p < 0.0001).

Next, we examined if the predicted *L. monocytogenes queT* riboswitch has gene regulatory activity *in vivo*. To this end, we inserted 43 nt of the 5′UTR of *queT* upstream of a green fluorescence protein (GFP) reporter gene and determined GFP expression in an *E. coli* strain that is unable to produce preQ_1_ by western blot or fluorescence measurement.^45^ The results show a strong reduction of GFP production upon addition of preQ_1_ (Fig. 3a–c). To examine whether the mode of action involves transcriptional or translational repression, we tested the expression of GFP mRNA by qPCR. We found increased rather than decreased transcript levels in the presence of preQ_1_ (Fig. 3d). Thus, we conclude that *Listeria queT* is regulated by a translational rather than a transcriptional riboswitch. Nevertheless, the observed significant increase of mRNA led us to speculate that binding of preQ_1_ might affect mRNA stability. To test whether this phenomenon also occurs with the native *queT* mRNA in *L. monocytogenes*, we performed qPCR following the addition of preQ_1_. However, we did not observe significant changes in *queT* mRNA abundance in the native context (Fig. 3e).

A possible explanation for the observed mRNA increase in *E. coli* could involve RNase E. Previous studies have demonstrated that RNase E, the primary mRNA degradation enzyme in *E. coli* and other bacteria, recognizes AU-rich sequences and can be inhibited by structural impediments, such as base pairing with antisense RNA or ribosome binding.^46^ In the *queT* riboswitch, the extended A-stretch might act as an RNase E cleavage site which could become inaccessible due to structural changes induced by preQ_1_ binding. Indeed, similar stabilization effects have been reported for other riboswitches, such as the guanidine III riboswitch^47^ and the SAM-II riboswitch,^48^ where ligand binding protects against RNase E cleavage. Since the GFP reporter system produces significantly larger amounts of mRNA than the native promoter in *L. monocytogenes* (Fig. S6), it is possible that any inhibitory effect of preQ_1_ on RNase E – *i.e.* a stabilizing effect on the mRNA – is more readily detectable in the reporter system than in the native context.

### Biophysical characterization of *L. monocytogenes queT* riboswitch

**Fig. 4:**
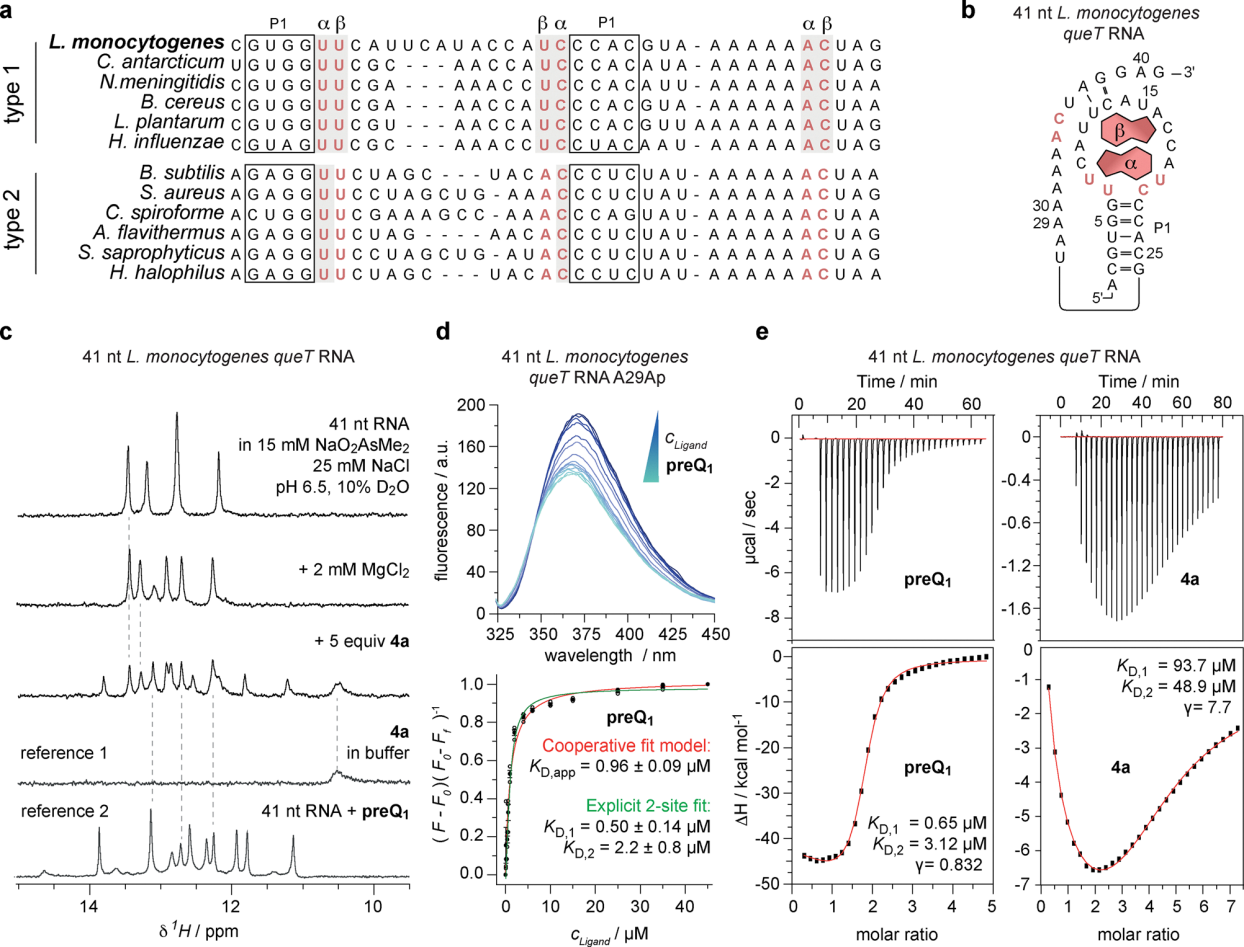
*L. monocytogenes queT* riboswitch binds two preQ_1_ molecules. (a) Sequence alignment of preQ_1_-I type 1 and type 2 sequences from different bacteria. The *L. monocytogenes queT* riboswitch is closest to type 1 sequences. Sequences were obtained from RNAcentral. Selected nucleotides interacting with the two ligand molecules (α, β) for type 1 RNA are shown in red,^7,24,52^ boxed nucleotides form the P1 stem. (b) Sequence and predicted secondary structure of the preQ_1_ riboswitch linked to *queT*. Nucleotides interacting with the two ligand molecules are shown in red. (c) ^1^H-NMR spectroscopy of 41 nt *queT* RNA shows significant changes in the imino proton chemical shift region which is consistent with the structural rigidification of a high-affinity RNA–ligand complex. (d) Affinity (*K*_D_) determination of preQ_1_ and *queT* RNA using a 2-aminopurine fluorescence assay; for details see main text. (e) Affinity (*K*_D_) determination of preQ_1_ and the biotin-preQ_1_ conjugate 4a, respectively, with *queT* RNA using isothermal titration calorimetry (ITC); for details see main text.

To further characterize the *queT* riboswitch, we performed sequence alignments with known class I riboswitches, thus classifying *L. monocytogenes queT* as a type 1 riboswitch (Fig. 4a). This group was recently found to cooperatively bind two ligand molecules in a stacked conformation.^24^ Secondary structure prediction of the 41 nt region upstream of the translational start codon showed the characteristic preQ_1_-I riboswitch fold (Fig. 4b).^7^

We then carried out a detailed analysis of ligand binding to the *queT* riboswitch. First, ^1^H NMR spectroscopic experiments were performed, allowing the identification of base pairs in RNA based on the detection of hydrogen bonds involving imino protons (*e.g.* Watson–Crick: N1–H of G paired to C, N3–H of U paired to A).^49,50^ An increase in the number of imino proton signals was observed when the 41 nt *queT* preQ_1_ RNA (Fig. 4c, 1st spectrum) was treated with Mg^2+^ (Fig. 4c, 2nd spectrum), and subsequently with the biotin-preQ_1_ conjugate 4a (Fig. 4c, 3rd spectrum). This is consistent with rigidification of a structurally flexible RNA through high-affinity binding of its cognate ligand. Moreover, the obtained signal pattern resembled the one obtained for the same RNA bound to non-functionalized preQ_1_ (Fig. 4c; ref. 2). Although some chemical shift deviations are observed (which are likely attributed to a loose interaction of the handle with the RNA fold), the binding mode of 4a and non-functionalized preQ_1_ appear similar.

Next, we determined the affinity of preQ_1_ to the 41 nt *queT* RNA, again using a 2-aminopurine modified variant which showed a fluorescence decrease upon titration with increasing amounts of preQ_1_ (Fig. 4d). A cooperative fit model gave an average *K*_D,av_ of 0.96 μM and a Job plot analysis (Fig. S7) indicated the binding of two non-functionalized preQ_1_ molecules. An advanced 2-site fit model^51^ allowed to estimate the individual affinities to be *K*_D,1_ = 0.5 μM and *K*_D,2_ = 2.2 μM (Fig. 4d). Subsequently, the findings were validated by isothermal titration calorimetry (ITC). The data was modelled using a “set of identical sites” model resulting in an average macroscopic *K*_D_ of 1.5 μM, and a 2 : 1 binding stoichiometry (Fig. S7). A “two-interdependent non-equivalent sites” model^24^ fitting provided two macroscopic dissociation constants (*K*_D,1_ = 0.65 μM and *K*_D,2_ = 3.12 μM; Fig. 4e, Fig. S8) that were in excellent agreement with the fluorescence data.

The biotin-preQ_1_ conjugate 4a exhibited an affinity one to two orders of magnitude lower compared to non-functionalized preQ_1_ (Fig. 4e and Fig. S9). Moreover, of particular interest was the observation that preQ_1_ binding occurred with slightly negative cooperativity (*y* = 0.8, Fig. 4e), while a robust positive cooperativity (*y* = 7.7, Fig. 4e) was observed for binding of 4a, meaning that the 4a occupation of the second site is facilitated by the pre-organization of the binding pocket induced by the first 4a molecule.

Together, our results demonstrate that the preQ_1_ transporter protein QueT in *L. monocytogenes* is controlled by a preQ_1_-I translational riboswitch that belongs to subgroup I, which is characterized by the simultaneous binding of two ligand molecules.

### Identification of other *Listeria* preQ_1_-binding mRNAs

**Fig. 5:**
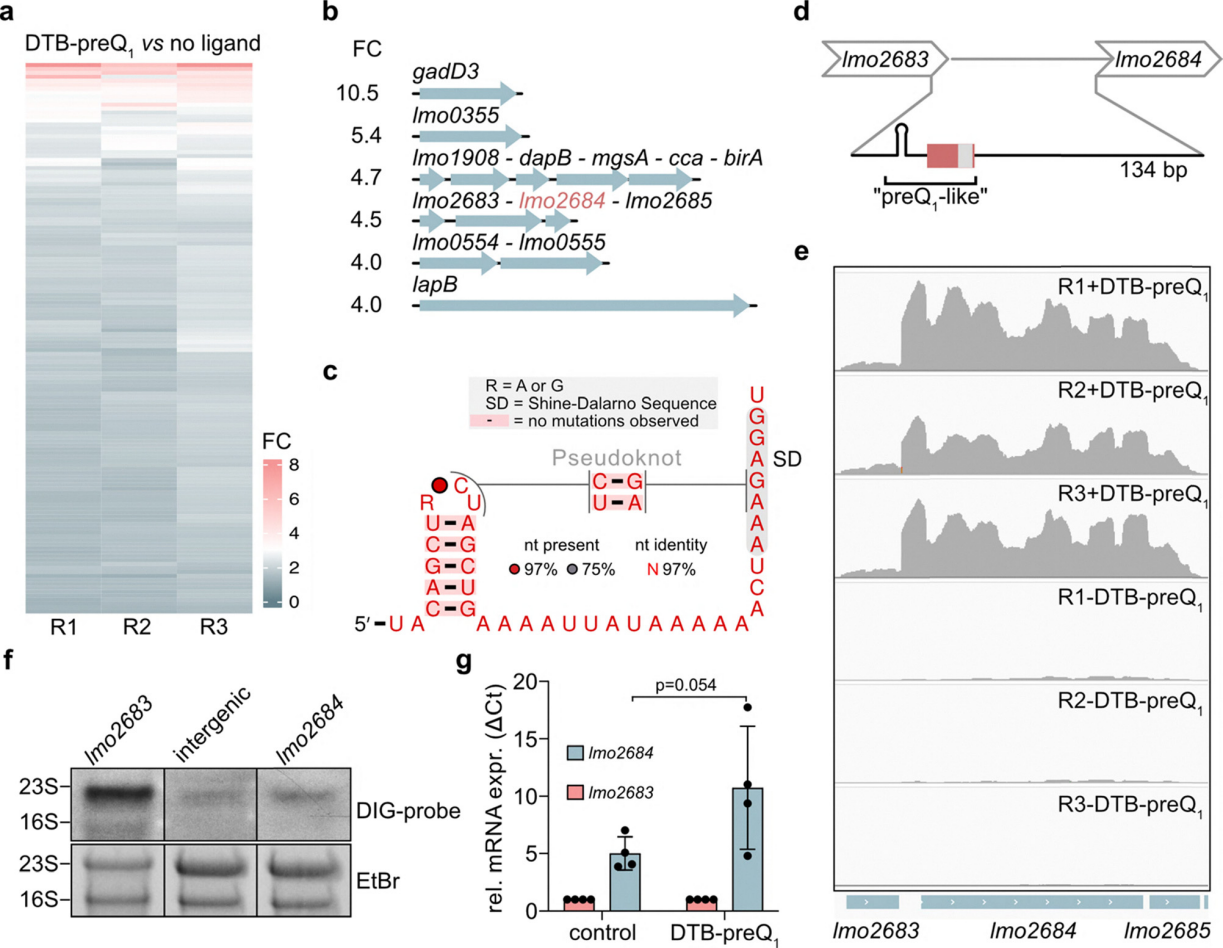
DTB-preQ_1_ (4b)-mediated enrichment of *L. monocytogenes* mRNAs. (a) Heatmap representing fold enrichment of mRNAs from DTB-preQ_1_-streptavidin pull-down experiments compared to no-ligand pull-down. Three biological replicates (R1, R2, R3) are shown. (b) Operon structure and fold enrichment (FC) of top 6 candidates. (c) Predicted secondary structure of *lmo2684* upstream region. (d) Schematic depiction of the intergenic region of *lmo2683–lmo2684*. Red box, conserved sequence, grey box, predicted Shine–Dalgarno sequence. (e) Integrated genome viewer (IGV) tracks of sequencing reads over the *lmo2683–lmo2685* operon from three replicates each (R1–R3) of pull-down reactions with or without DTB-preQ_1_ (4b). f, Northern blot analysis of *L. monocytogenes* total RNA using DIG-labelled probes that hybridize to the indicated sequences. The positions of 23S and 16S rRNA bands are denoted. Images of the corresponding ethidium bromide-stained gels (EtBr) are shown. (g) Results of qPCR analysis of cDNA reverse transcribed with a *lmo2685*-specific primer in the presence or absence (control) of DTB-preQ_1_ (4b). 2^Δ*C*_t_^ values of *lmo2683* and *lmo2684* were calculated. Values were normalized to *lmo2683*. Mean ±SD of 4 experiments is shown. Unpaired t-test was conducted for statistical significance testing.

Although *queT* is the only predicted preQ_1_ riboswitch in *L. monocytogenes*,^7,23^ we sought to explore the possibility to identify new preQ_1_-binding RNAs in this organism. To this end, we combined the streptavidin pull-down strategy with deep sequencing. Enriched sequences in DTB-preQ_1_ (4b) pull-down samples, compared to no-ligand pull-downs, were determined using an nf-core workflow (see Methods). PCA plots showed clear segregation of ligand and negative control samples (Fig. S10). Approximately 140 peaks enriched in DTB-preQ_1_ (4b) pull-down compared to no-ligand samples were detected across all three replicates (Fig. S10), with the top six candidates showing 4-10-fold enrichment (Fig. 5a and Table S1). These candidates mapped to multigene operons or single genes, respectively (Fig. 5b).

We then subjected the top candidates to sequence homology analysis using the RFAM database which resulted in two hits with preQ_1_ riboswitches. These corresponded to the 5′UTR of *lmo2684* and a sequence located in the coding region of the *dnaG* gene. However, conservation was only detected for the sequence part corresponding to the A-stretch and surrounding nucleotides of canonical preQ_1_ riboswitches but not for the P1 stem and loop region that are necessary for ligand binding (see Fig. 4b). Local structure prediction revealed a stem loop starting 12 nt upstream of the conserved region (Fig. S11a and Fig. 5c). Even though the sequence of this stem loop structure does not resemble canonical preQ_1_ riboswitches, there is similarity in the predicted secondary structure (Fig. 5c and Fig. S11b). Further homology searches found this sequence to be conserved in several *Listeria* species (Fig. S11c). We therefore decided to examine the *lmo2684* upstream region (hereafter termed “preQ_1_-like”) in more detail. *Lmo2684* is the second gene in a three-gene operon encoding phosphotransferase system (PTS) cellobiose transporter subunits IIA (*lmo2685*), IIB (*lmo2683*) and IIC (*lmo2684*) (Fig. 5b).^53^

The preQ_1_-like sequence lies in the intergenic region between *lmo2683* and *lmo2684* (Fig. 5d). Interestingly, even though the three genes are classified as a transcriptional unit, very few sequencing reads covered the *lmo2683* gene (Fig. 5e) suggesting that the upstream gene might not be part of the transcriptional unit as annotated. However, northern blot analysis with probes specific to *lmo2683*, *lmo2684* or the intergenic region between the two revealed that all three probes detected a band migrating slightly faster than the 23S rRNA band (2900 nt; Fig. 5f). These results indicate that *lmo2683* is indeed part of a single polycistronic mRNA (predicted size ∼2100 nt) encompassing all three genes of the operon.

A conceivable explanation for the observed loss of read coverage of *lmo2683* could be a block of reverse transcription during library preparation caused by ligand binding. To test this possibility, we conducted qPCR analyses with cDNA generated from total *L. monocytogenes* RNA that was either incubated with DTB-preQ_1_ (4b) or water and subsequently reverse transcribed using a primer complementary to the 3′ region of the operon. The qPCR primer pairs were located either in *lmo2683* or in *lmo2684*. The results show that preincubation of the RNA with DTB-preQ_1_ (4b) resulted in increased amplification of the *lmo2684* region compared to *lmo2683* (Fig. 5g). Together these results provide further evidence for a direct interaction of the ligand with the intergenic region between *lmo2683* and *lmo2684*.

We next attempted to detect preQ_1_ binding to the preQ_1_-like sequence using NMR spectroscopy but found no high affinity association (Fig. S12). Although the 40 nt RNA was chosen based on the secondary structure predictions, this fragment might not comprise the actual binding-competent motif. Further attempts with 5′ and/or 3′ sequence extensions (of about 10 to 15 nt) flanking the 40 nt core RNA were made, but again, no preQ_1_ binding was detectable (data not shown). It is important to note, however, that the RNA synthesis length limitation of ∼65 nt hindered further NMR analyses. Therefore, this negative result does not definitively rule out the possibility that preQ_1_ binds to a slightly different or larger sequence within the *lmo2683*–*lmo2684* intergenic region.

### preQ_1_-mediated regulatory activity of the *lmo2683*–*lmo2684* intergenic region

**Fig. 6:**
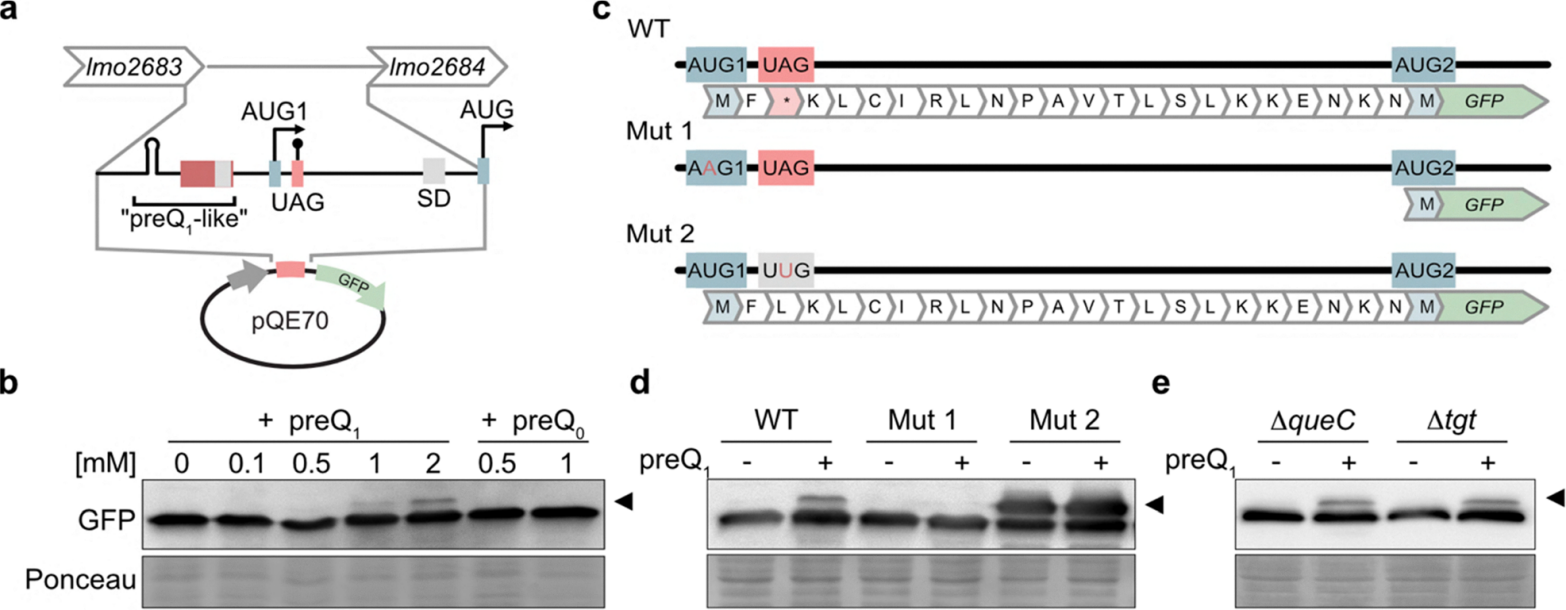
Functional analysis of the preQ_1_-like sequence in the *lmo2683–lmo2684* intergenic region. (a) Putative functionally important elements in the *lmo2684* upstream region. The entire intergenic sequence was cloned into the pQE70 expression vector upstream of the GFP reporter gene. Red box, conserved sequence; SD and grey boxes, putative Shine–Dalgarno sequences; AUG_1_ and UAG, putative upstream start and stop codons; AUG, canonical translational start codon of *lmo2684*. (b) Western blot of *E. coli* Δ*queC* cells transformed with the WT reporter construct (c) and treated with increasing preQ_1_ or preQ_0_ concentrations as indicated. The membrane was detected with an α-GFP antibody. The slower migrating band is indicated by an arrowhead. Equal loading was confirmed by Ponceau S staining of the membrane. (c) Schematic representation of mutations introduced into the upstream sequence of *lmo2684* in the pQE70-GFP reporter construct. WT, control sequence; Mut1, mutation of the putative upstream start codon AUG_1_; Mut2, mutation of the AUG_1_-adjacent stop codon. Corresponding protein translations are shown. The star signifies a stop codon and AUG_2_ marks the canonical GFP start codon. (d) Western blot of *E. coli ΔqueC* bacteria transformed with WT or mutated reporter constructs (c) and treated or not with 1 mM preQ_1_. (e) Western blot of *E. coli ΔqueC* or *Δtgt* bacteria transformed with WT reporter construct (c) and treated or not with 1 mM preQ_1_.

To investigate whether the *lmo2683–lmo2684* intergenic region harbours preQ_1_-dependent regulatory activity, we cloned the entire intergenic region into the GFP reporter plasmid (Fig. 6a) and monitored its effects on GFP production in the preQ_1_-deficient *E. coli* strain JW0434 (*ΔqueC*) in conditions with or without preQ_1_ or its inactive precursor preQ_0_. Intriguingly, while the addition of preQ_1_ did not affect GFP translation efficiency, we observed the emergence of an additional, slower-migrating band in the presence of preQ_1_. The intensity of this band correlated with the preQ_1_ concentration (Fig. 6b). In contrast, the addition of preQ_0_ did not result in the appearance of the larger band. Even though the used ligand concentrations are likely above physiological levels, it is important to consider that mRNA expression from the reporter construct is several orders of magnitude stronger than the native mRNA level (Fig. S6) and might therefore require higher preQ_1_ concentrations for saturation.

The data suggested that an alternative translational start codon upstream of the canonical one might be used in the presence of preQ_1_. Inspection of the intergenic region of *lmo2683–lmo2684* revealed the presence of an upstream AUG that was in frame with the GFP open reading frame in the reporter construct. However, initiation from this start codon would terminate in a nearby stop codon (Fig. 6c and Fig. S11c). In order to explain the observed larger GFP band, translation from the most 5′ located AUG (AUG_1_) would necessitate read-through of the neighbouring amber (UAG) stop codon. To test this hypothesis, we mutated either AUG_1_ or the adjacent stop codon (Fig. 6c). Intriguingly, AUG_1_ mutation abolished the larger GFP band, while mutation of the adjacent stop codon caused strong production of the larger band independently of the presence of preQ_1_ (Fig. 6d). These results suggest that selection of AUG_1_ is not dependent on preQ_1_ and occurs frequently, while stop codon read-through is dependent on preQ_1_. To rule out the possibility that the latter was due to a mechanism involving stop codon suppression by a Q-containing tRNA, we performed the reporter assay in an *E. coli* strain lacking tRNA guanine transglycosylase (*Δtgt*) which is not able to generate Q-modified tRNAs.^18,54^ The larger band was again detected arguing against a role for Q-containing tRNAs (Fig. 6e).

To determine whether the putative alternative start and stop codons are unique to the bacterial strain used in our study or are more broadly conserved, we conducted sequence homology analyses across 35 *L. monocytogenes* strains and two additional *Listeria* species that contain the preQ_1_-like sequence motif. These analyses revealed that the *lmo2683–2684* intergenic region, including AUG_1_ and the adjacent stop codon, exhibits a high degree of sequence conservation. Interestingly, rather than serving as an alternative translation start site for the canonical *lmo2684* gene, the data suggest that AUG_1_ likely marks an upstream open reading frame (uORF). This uORF could give rise to a 21 or 23-amino-acid peptide (depending on the specific strain) in the presence of preQ_1_ (Fig. S11c and d). At present, the functional role of such a peptide in *Listeria* remains unclear.

In summary, our findings suggest that the identified preQ_1_-like sequence in the *lmo2683–2684* intergenic region does not function through a typical riboswitch mechanism that regulates transcription or translation efficiency. Instead, the data indicate that preQ_1_ modulates stop-codon read-through in this region thereby enabling uORF translation.

## Conclusions

The identification of naturally occurring RNA aptamers has traditionally been governed by bioinformatics analyses and genetic screens. In this work, we introduce the possibility to identify such regulatory elements using affinity purification with a modified ligand, such as DTB-preQ_1_ (4b). As proof of principle, we demonstrated efficient enrichment of the previously uncharacterized *queT* mRNA from *L. monocytogenes*, which contains a predicted preQ_1_ class I riboswitch in its 5′UTR. We showed that this riboswitch binds two preQ_1_ molecules and negatively regulates *queT* translation in the presence of the ligand. Furthermore, we show that combining the affinity pull-down with deep sequencing enables the enrichment of preQ_1_-binding sequences from *Listeria* total RNA.

Notably, we discovered preQ_1_-dependent regulation of the use of a short reading frame in the upstream region of *lmo2684* by enabling stop codon read-through. The significance of our results is twofold: firstly, the experimental approach to discover new preQ_1_-binding RNAs enabled the detection of a sequence that had escaped detection by previous bioinformatics methods, thus providing a viable complementary strategy in the search for small ligand-binding RNAs. Secondly, the observed regulatory function of preQ_1_ in the context of the *lmo2684* upstream region is novel, as it involves control of the use of an uORF rather than regulation of translation initiation efficiency of the main ORF. Further studies are needed to elucidate the exact mode of preQ_1_–RNA interaction in this context.

It is interesting to note that the identified preQ_1_ target is a gene that has no known role in the synthesis or salvage of Q or its precursors. Instead, it is part of a cellobiose phosphotransferase system. Q hypermodification by mannose or galactose has been shown for eukaryotes,^55^ raising the theoretical possibility for a functional link between preQ_1_ and sugar metabolism. However, no such hypermodification has yet been found in bacteria, so the potential physiological connection remains unclear.

## Experimental

### Synthesis of DTB-preQ_1_ conjugates

The synthesis of DTB-preQ_1_ conjugates is described in the SI (Fig. S1–S4).

### Chemical RNA synthesis

RNA sequences were synthesized according to established procedures using the 2′-OTBMDS phosphoramidite approach on controlled-pore glass solid supports at a 2 μmol scale.^56^ RNAs were deprotected, purified and analyzed as recently described.^57^ See Table S2 for sequences and mass spectrometric data.

### 2ApFold kinetic assays

2-Aminopurine-labeled preQ_1_ RNA was dissolved in binding buffer (100 mM KCl, 2 mM MgCl_2_, 50 mM MOPS pH 7.5) to give a 1.0 μM solution. The RNA was refolded by heating to 90 °C for 2 min and cooling on ice for further 2 min. A dilution series of four different ligand (preQ_1_ or 4a) concentrations was generated (2, 4, 12 and 20 μM in H_2_O). Fluorescence measurements were performed at 25 °C on a Cary Eclipse fluorescence spectrometer equipped with a Peltier element and an RX2000 stopped-flow apparatus (Applied Photophysics Ltd.). RNA and ligand samples were allowed to pre-equilibrate at 25 °C for 15 min before the measurement. Upon 1 : 1 mixing in the stopped-flow cell (250 μL), the change in fluorescent signal was monitored over the course of 700 s. Entry and exit slit widths were 10 nm; 308 and 372 nm were chosen as the excitation and emission wavelengths, respectively. Detector voltage was 600 V. Measurements were performed in triplicate. The change in fluorescence signal *F* was plotted *versus* time and fitted to *F* = *A* × (1 − e^−*k*_obs_ × *t*^). The *k*_obs_ values were plotted against ligand concentration and linear regression to *k*_obs_ = *k*_on_ × *c*_L_ + *d* provided on rates, where *c*_L_ is the ligand concentration. Off rates were calculated by *k*_off_ = *k*_on_ × *K*_D_, where *K*_D_ is the dissociation constant determined by the equilibrium 2ApFold measurements described below. Data analysis was performed in Origin 2020 (OriginLab).

### 2ApFold assay for determination of binding affinity

2-Aminopurine-labeled preQ_1_ RNA was dissolved in binding buffer (100 mM KCl, 2 mM MgCl_2_, 50 mM MOPS pH 7.5) to give 1 mL of a 0.5 μM solution. The RNA was refolded by heating to 90 °C for 2 min and cooling on ice for further 2 min. Fluorescence measurements were performed at 25 °C on a Cary Eclipse fluorescence spectrometer equipped with a Peltier element and a magnetic stirring unit. RNA samples were allowed to pre-equilibrate at 25 °C for 15 min before the measurement. Initial Fluorescence (in the absence of ligand) was measured (325–450 nm). Entry and exit slit widths were 5 or 10 nm; 308 was chosen as excitation wavelength. 1 μL of a ligand (preQ_1_ or 4a) stock was added in each titration step to give the total ligand concentrations (0, 0.1, 0.2, 0.4 0.6, 1.0, 2.0, 4.0, 6.0, 10.0, 15.0, 25.0, 35.0, 45.0 μM for *Tte vs.*4a and *queT vs.* preQ_1_; 0, 0.1, 0.2, 0.4 0.6, 1.0, 2.0, 4.0, 6.0, 10.0, 20.0, 30.0 for *Tte vs.* preQ_1_). After each addition, the stirred solution was allowed to equilibrate for 20 min, after which the fluorescence data was recorded (325-450 nm). Measurements were performed as three independent replicates. The fluorescence spectra were integrated between 350 and 450 nm and normalized by (*F*–*F*_0_)(*F*_f_ − *F*_0_)^−1^, where *F*_0_ is the initial fluorescence and *F*_f_ is the final fluorescence. The normalized fluorescence was plotted against ligand concentration and fitted to (*F* − *F*_0_)(*F*_f_ − *F*_0_)^−1^ = (*K*_D_ + *c*_RNA_ + *c*_L_ – ((*K*_D_ + *c*_RNA_ + *c*_L_)^2^ − 4 × *c*_RNA_ × *c*_L_)^0.5^)/(2 × *c*_RNA_ – *d*), where *c*_RNA_ is the RNA concentration, *c*_*L*_ is the ligand concentration to obtain *K*_D_ values. For the cooperative and the 2-site model the data was fitted to (*F* − *F*_0_) (*F*_f_ − *F*_0_)^−1^ = (*d × c*_L_^*n*^)/(*K*_D_^*n*^ + *c*_L_^*n*^) and *F* − *F*_0_ (*F*_f_ − *F*_0_)^−1^ = *d* × (*K*_D1_ × *c*_L_ + 2 *× K*_D1_ × *K*_D2_ × *c*_L_^2^)/(1 *+ K*_D1_ × *c*_L_ + *K*_D1_ × *K*_D2_ × *c*_L_^2^), respectively, where *n* is the Hill coefficient and *d* is a free fit parameter. Data analysis was performed in Origin 2020 (OriginLab).

### Job plot analysis

A dilution series of 2-aminopurine-labeled preQ_1_-binding RNA in binding buffer was prepared (0, 2, 4, 6, 8, 10, 12, 14, 16, 18, 20 μM in 120 μL each). The RNA was refolded by heating to 90 °C for 2 min and cooling on ice for further 2 min. A fluorescence scan was performed (325–450 nm; for conditions and parameters, see 2ApFold assay for determination of binding affinity). To these solutions was added 1 μL of ligand stock so that *c*_RNA_ + *c*_L_ = 20 μM is fulfilled. After 20 minutes the spectra were again recorded and integrated between 350 and 450 nm. The fluorescence after ligand addition *F*_F_ was subtracted from the initial value *F*_0_ for each set. Measurements were performed as three independent replicates. *F*_0_ − *F*_F_ was plotted against *c*_RNA_ (*c*_RNA_ + *c*_L_)^−1^. Tangents were fitted to either side of the peak-shaped curve and intersected to get the maximum fluorescence. The *x*-value at the maximum of around 0.33 is indicative of 2 : 1 binding. Data analysis was performed in Origin 2020 (OriginLab).

### Isothermal titration calorimetry

ITC measurements were performed on a MicroCal iTC200 instrument. Lyophilized RNA was dissolved in binding buffer (100 mM KCl, 2 mM MgCl_2_, 50 mM MOPS pH 7.5) and refolded by heating to 90 °C for 2 min and cooling on ice for further 2 min. Ligands (dissolved in binding buffer) were in the syringe, RNA in the cell. For measurements fitted by the “set of identical sites” model, measurements were performed at 25 °C over 20 injections (injection volume: 2 μL) and a spacing of 150 s. Fitting was done by the software included with the ITC. In case of measurements performed for the “two-interdependent non-equivalent sites” model, data were recorded at 37 °C over 30 injections (1.3 μL) with a spacing of 150 s. The concentrations of RNA and ligand were as follows: *queT vs.* preQ_1_ at 25 °C: 73 μM RNA, 1.84 mM ligand; *queT vs.* preQ_1_ at 37 °C: 48 μM RNA 1.15 mM Ligand. *queT vs.*4a at 37 °C: 63 μM RNA, 2.25 mM ligand. The data was fitted with a recently published Python program based on binding polynomial theory.^24^

### NMR imino spectra

Lyophilized RNA samples (as sodium salts) were dissolved in 500 μL NMR buffer (15 mM sodium cacodylate, 25 mM NaCl, pH 6.5, 10% D_2_O, 0.01% NaN_3_) to give a final concentration of c(RNA) of 0.15 mM. The RNA was heated to 90 °C for 2 min and allowed to cool to room temperature for further 10 min. Spectra were recorded at 25 °C before and after addition of MgCl_2_ (2 mM) and ligand (preQ_1_ or 4a). All NMR experiments were conducted on a Bruker 600 MHz Avance II + NMR or a 700 MHz Avance Neo NM both equipped with a Prodigy TCI probe.

### Bacterial strains and growth conditions

*L. monocytogenes serotype* 4b (NCTC 11994) and *E. coli* strains JW0434 (*ΔqueC*) and JW0396 (*Δtgt*)^54^ were used. *E. coli* was cultured in Luria-Bertani (LB) media, and *L. monocytogenes* was grown in LB or brain heart infusion (BHI, Roth) agar plates or liquid media at 37° with shaking. When necessary, 100 μg mL^−1^ ampicillin and 400 μg mL^−1^ kanamycin was added to the media.

### RNA isolation

Total RNA was isolated from bacteria using the hot phenol RNA isolation protocol essentially as described in ref. 58. Typically, 8 mL of a bacterial culture at OD_600_ = 0.2 was used and the resulting RNA was dissolved in 50 μL RNase-free water.

### *In vitro* transcription

To generate *queT* and *bglA* RNA fragments, transcription templates were generated by PCR with cDNA obtained from *L. monocytogenes* RNA using the GoScript™ Reverse Transcription System (Promega) with random hexamers (Promega), and target-specific primers (see Table S3 for primer sequences). The PCR product was cloned into the pGEM®-T vector (Promega). After linearization the obtained plasmid was used as template for *in vitro* transcription using the HiScribe T7 High Yield RNA Synthesis Kit (New England Biolabs) according to the manufacturer's instructions. Products were digested with DNase I (2000 U mL^−1^, NEB), purified by phenol-chloroform-isoamylalkohol (24 : 23 : 1) extraction (Roth), precipitated with 2.5 volumes ethanol absolute and 1/10 volume 3M NaOAc (pH 5.2) and dissolved in 50 μL RNase-free water.

### Streptavidin pull-down and on-bead cDNA synthesis

All steps were performed in 1.5 mL LoBind tubes (Eppendorf). 200 μg M-280 streptavidin Dynabeads (Invitrogen) were prepared for RNA processing as recommended by the manufacturer followed by resuspension in 80 μL 1 × B&W buffer (5 mM Tris-HCl pH 7.5, 0.5 mM EDTA, 1 M NaCl). Five nmol DTB-preQ_1_ (4b)(5.7 mM stock in water or DMSO) or DTB-alkyne (3b) (5.7 stock in water or DMSO) was added, and the beads were incubated at room temperature for 15 min with gentle rotation. To remove unbound ligand, the beads were washed twice with 100 μL 1 × B&W followed by 100 μL LBB (2 mM MgCl_2_, 100 mM KCl, 50 mM MOPS pH 6.0). In parallel, 2 μg total RNA or 10 ng *in vitro* transcript were mixed with 40 μL LBB, heat denatured (90 °C) for 2 min and refolded by cooling to 25 °C over 10 min in a PCR machine and subsequently incubated with the DTB-preQ_1_-bound beads for 30 min at room temperature with rotation. For competition assays with non-modified preQ_1_ (Fig. 2f), the RNA was preincubated with 500 nmol preQ_1_ prior to incubation with the DTB-preQ_1_ bound beads. Unbound RNA was removed by placing the tubes into a magnetic stand, and beads were washed three times in Wash Buffer 1 (15 mM Tris-HCL pH 7.5, 150 mM NaCl, 0.1% NP-40) and three times in Wash Buffer 2 (15 mM Tris-HCl pH 7.5). The bound RNA was reverse transcribed into cDNA directly on the beads using random hexamer primers and the GoScript™ Reverse Transcription System (Promega). Additionally, 2 μg total RNA were directly transcribed into cDNA as an input control.

### qPCR

To quantify DTB-preQ_1_-enriched RNA or to assess mRNA expression levels, cDNA was subjected to qPCR analysis with three technical replicates using the Luna ®Universial qPCR reagent (New England Biolabs) according to the manufacturer's instructions in a QuantStudio 3 Real-Time PCR System (Applied Biosystems). Primer sequences are shown in Table S3. The *C*_t_ mean values of the three replicates were converted to 2^−*C*_t_^ values. The pull-down was quantified using an input control corresponding to an aliquot of the RNA used for the respective pulldown experiment. The ratio between the mRNA levels in the input control and the pulldown sample was calculated and normalized against the respective negative control (*e.g.* no ligand pull-down) to determine the “relative enrichment”.

### Northern blot analysis

Total RNA (8 μg) from *L. monocytogenes* was loaded onto a 1.2% agarose gel containing 1.85% (w/vol) formaldehyde and subsequently blotted onto a Hybond-N+ membrane (Cytiva) and hybridized with DIG-labeled probes (Roche) generated by PCR with primers listed in Table S3.

### Library generation and deep sequencing

For deep sequencing analysis of streptavidin-enriched RNAs, sequencing libraries were generated of three replicates each of DTB-preQ_1_ and mock (no ligand) pull-down reactions using the CORALL RNA-Seq V2 Library Prep kit for low input material (Lexogen) according to the manufacturer's instructions. Briefly, pull-downs were performed with 15 μg RNA, 8.7 nmol DTB-preQ_1_ (4b) ligand and 20 μL M-280 Streptavidin Dynabeads as described above. After the final washing step, the beads were resuspended in 10 μL RNase-free water. Next, a library was prepared using the “short insert sizes” protocol of the CORALL RNA-Seq V2 Library Prep (Lexogen). Since initially the RNA was still bound to the beads, the Displacement Stop Primer hybridization and reverse transcription were performed on the beads. Then, the beads were discarded and the supernatant was used for the subsequent steps according to the manufacturer's instructions. After quality control, the libraries were pooled and sequenced using an Illumina MiSeq system with a depth of 24–30 million reads.

### Riboswitch activity assay in *E. coli*

To evaluate translational repression activity, a 43 bp region upstream of the annotated *L. monocytogenes queT* translational start codon comprising the predicted riboswitch sequence or the 134 bp long region between the coding regions of *lmo2683* and *lmo2684*, respectively (Fig. 5d), was inserted into pQE70 (Qiagen) upstream of a GFP reporter gene.^45^*E. coli* JW0434, a *queC* deletion strain that is unable to produce preQ_1_,^54^ was transformed with these constructs. At OD_600_ of ∼0.5, 1 mM preQ_1_ was added and the bacteria were allowed to grow for 4–6 hours before taking 50 μL of bacterial culture for western blot analysis and 1.5 mL for RNA isolation. Alternatively, bacteria were grown in 96-well plates and GFP levels were determined by measuring fluorescence and OD_600_ values at different intervals after preQ_1_ addition using a CLARIOstar Plus (BMG Labtech) plate reader. Fluorescence values were blank-corrected (LB media) and normalized to show a range of 0 to 1.

### Western blot analysis

Bacterial pellets of 50 μL culture were subjected to SDS-PAGE and immunoblotting using standard procedures. Antibody incubation overnight with mouse α-GFP antibody (1 : 10 000, Roche) was followed by HRP-conjugated secondary antibody (1 : 10 000, Sigma). Signal development and detection were done using the ECL system (Abcam) and a Fusion-SL 3500-WL instrument (Vilber Lourmat). Raw western blot images are shown in Fig. S13.

### Data analysis

Sequencing reads were filtered for rRNA using SortMeRNA (v.4.3.4). Filtered reads were processed by the nf-core/chipseq pipeline (version 2.1.0) with default parameters (10.5281/zenodo.3240506).^59^ Raw reads were quality-checked using FastQC (v.0.12.1), followed by adapter and quality trimming with Cutadapt (version 3.4). Reads were then aligned to the *L. monocytogenes* serotype 4b str. NCTC 11994 genome (GCF_002156185.1_ASM215618v1_genomic.fna) using BWA (0.7.18-r1243-dirty).^60^ Duplicates were marked with Picard MarkDuplicates (v. 3.2.0-1-g3948afb6b), and mapping statistics were collected using Samtools (v.1.17; Table S4). Peaks for preQ_1_ binding were called using MACS3 (v3.0.1) against mock pull-down controls, and annotated with HOMER (v.4.11). Mean values of enrichment factors of three replicates were calculated.

### Multiple sequence alignments

The alignment in Fig. 4a was generated using the ClustalW algorithm and manually curated. Sequences for the alignment in Fig. 4a are shown in Table S5.

### Homology analysis of preQ_1_ candidates

The search for potential preQ_1_-riboswitch-like motifs in pull-down candidates is described in detail in SI.

## Author contributions

Conceptualization: A. L., R. M. Investigation: M. H., L. F., C. M. Formal analysis: M. H., I. B., L. F., C. M., C. A. V. H Funding acquisition: A. L., R. M. Visualization: M. H., L. F., I. B., C. A. V. H, R. M., A. L., writing – original draft: M. H., A. L., R. M. Writing – review & editing: M. H., I. B., L. F., C. M., C. A. V. H, I. H., R. M., A. L.

## Conflicts of interest

There are no conflicts to declare.

## Supplementary Material

CB-006-D5CB00102A-s001

## Data Availability

The data supporting this article have been included as part of the supplementary information (SI). Supplementary information is available. See DOI: https://doi.org/10.1039/d5cb00102a. RNA sequencing data have been deposited at the Sequence Read Archive (SRA) under Bioproject number PRJNA1255589.
