# Supplementary material for: Experimental identification of preQ1-binding RNAs in the pathogenic bacterium Listeria monocytogenes
Source: RSC Chem Biol. 2025 Oct 14;6(12):1867–78. doi: 10.1039/d5cb00102a (PMC12519234; doi:10.1039/d5cb00102a)
Supplement: CB-006-D5CB00102A-s001 [file CB-006-D5CB00102A-s001.pdf]

Supplementary Information (SI) for RSC Chemical Biology.
This journal is © The Royal Society of Chemistry 2025

SUPPORTING INFORMATION

Experimental identification of preQ$_1$-binding RNAs in the pathogenic bacterium *Listeria monocytogenes*

Malou Hanisch,[a] Laurin Flemmich,[b] Christoph Mitteregger,[b] Ingo Bauer,[a] Cristian A. Velandia-Huerto,[c,d] Ivo Hofacker,[c,e] Ronald Micura,*[b] and Alexandra Lusser *[a]

[a.] Institute of Molecular Biology, Biocenter, Medical University of Innsbruck, Innrain 80-82, 6020 Innsbruck, Austria.
[b.] Institute of Organic Chemistry, Center for Molecular Biosciences Innsbruck, University of Innsbruck, Innrain 80-82, 6020 Innsbruck, Austria.
[c.] Department of Theoretical Chemistry, University of Vienna, Währinger Straße 17, 1090 Vienna, Austria
[d.] Center for Anatomy and Cell biology, Medical University of Vienna, Schwarzspanierstraße 17, 1090 Vienna, Austria
[e.] *Research Group Bioinformatics and Computational Biology, University of Vienna, Währinger Straße 29, 1090 Vienna, Austria*

*To whom correspondence should be addressed:

E-mail: ronald.micura@uibk.ac.at
E-mail: alexandra.lusser@i-med.ac.at

Table of contents

1. SUPPORTING INFORMATION METHODS 3

1.1. **Synthetic procedures** 3

1.2. **Homology analysis of preQ$_1$ candidates** 4

2. SUPPORTING FIGURES 6

Supporting Fig.S1 6

Supporting Fig. S2 7

Supporting Fig. S3 8

Supporting Fig. S4 9

Supporting Fig. S5 10

Supporting Fig. S6 11

Supporting Fig. S7 12

Supporting Fig. S8 13

Supporting Fig. S9 14

Supporting Fig. S10 15

Supporting Fig. S11 16

Supporting Fig. S12 17

Supporting Fig. S13 18

3. SUPPORTING TABLES 19

Supporting Table S1 19

Supporting Table S2 20

Supporting Table S3 20

Supporting Table S4 21

Supporting Table S5 21

4. SUPPORTING REFERENCES 22

1. Supporting Information Methods

1.1. Synthetic procedures

Synthesis of DTB-preQ$_1$ conjugates

General. Chemical reagents and solvents were purchased from commercial suppliers (Sigma-Aldrich, Jena Bioscience) and used without further purification. Dry solvents were used for all non-aqueous reactions, which were carried out under argon atmosphere. Analytical thin-layer chromatography (TLC) was performed on Machery-Nagel Polygram SIL G/UV254 plates. Reversed-phase chromatography was performed on an ÄKTAprime plus instrumentation using a prepacked Götech LiChroprep® RP-18 (40–63 µm) column. ^{1}H, and ^{13}C NMR spectra were recorded on Bruker Avance 400 MHz spectrometer. Chemical shifts (δ) are reported relative to tetramethylsilane (TMS) and referenced to the residual proton or carbon signal of the deuterated solvent: methanol-d$_4$ (3.31) for ^{1}H NMR; methanol-d$_4$ (49.00) for ^{13}C NMR spectra. ^{1}H and ^{13}C assignments are based on COSY, HSQC, and HMBC experiments. ESI-MS experiments were performed on a Thermo Fisher Qexactive Classic. Samples were analyzed in the positive-ion mode. LC-MS data was recorded on a Shimadzu LC-2020C 3D RoHS – Prominence-i.

7-(*N*-2'-azidoethyl)aminomethyl-7-deazaguanine trifluoroacetic acid salt (2). Compound 2 (68.0 mg, 382 µmol) was prepared according to the general procedure for reductive aminations described in reference[1]. The crude solid was acidified by trifluoracetic acid (10%) and purified by reversed-phase chromatography (eluent A: aqueous trifluoroacetic acid (0.1%); eluent B: acetonitrile, linear gradient: 0-20% B in 350 mL, flow: 3 mL min^{-1}) to give **2** (131 mg, 95%) as an off-white solid. TLC: 20 % MeOH in CH_2Cl_2, R_f : 0.58 (free-base amine). HR-ESI-MS (m/z): [M+H]$^+$ found: 249.1204; [M+H]$^+$ calculated: 249.1207. ^{1}H-NMR (400 MHz, MeOH-*d$_4$*): δ 6.90 (s, 1H, *H*C(8)), 4.32 (s, 1H, H_2C-C(7)), 3.75 (t, J_{HH} = 5.6 Hz, 2H, *H*C(1')), 3.23 (t, J_{HH} = 5.6 Hz, 2H, *H*C(2')) ppm. ^{13}C-NMR (100 MHz, MeOH-*d$_4$*): δ 161.9 C(6), 161.5 (CF_3COO^-, q, J_{CF} = 35 Hz) 154.1 *C*(2) 150.0 *C*(4),119.9 *C*(8), 117.4 (*C*F$_3$COO-, q, J_{CF} = 195 Hz), 109.9 & 100.0 *C*(5) & *C*(7), 48.5 *C*(2'), 46.6 *C*(1'), 44.6 *C*H$_2$C(7) ppm (Supporting Fig. S1).

***N*-(16-(1-(3-(((2-(λ^2-azaneyl)-4-oxo-4,7-dihydro-3*H*-pyrrolo[2,3-*d*]pyrimidin-5-yl)methyl)amino)propyl)-1*H*-1,2,3-triazol-4-yl)-3,6,7,10,11,14-hexaoxahexadecyl)-5-((3aS,4S,6aR)-2-oxohexa-hydro-1*H*-thieno[3,4-*d*]imidazol-4-yl)pentanamide trifluoroacetate salt (4a).** To a solution of **2** (10.0 mg, 27.6 µmol, 1.3 eq) in a mixture of acetonitrile and water (1:1, v:v, 1.0 mL) was added 6-((4*S*)-2-oxohexahydro-1*H*-thieno[3,4-*d*]imidazol-4-yl)-*N*-(3,6,9,12-tetraoxa-penta-dec-14-yn-1-yl)hexanamide (10 mg, 21.2 µmol, 1 eq) and copper sulfate (21.0 mg, 84.9 µmol, 4 eq) pre-dissolved in a minimal amount of water. After addition of ascorbic acid (75.0 mg, 425 µmol, 20 eq), the reaction was allowed to proceed for 3 h. The volatiles were removed in vacuo and the crude product re-dissolved in aqueous trifluoracetic acid (0.1%), containing acetonitrile (10%, v:v) and filtered. The filtrate was purified by reversed-phase chromatography (eluent A: aqueous trifluoroacetic acid (0.1%); eluent B: acetonitrile, linear gradient: 0-30% B in 400 mL, flow: 5.0 mL min^{-1}) to give **4a** (13.2 mg, 76%) as an off-white solid. HR-ESI-MS (m/z): [M+H]$^+$ found: 706.3455; [M+H]$^+$ calculated: 706.3453. See Supporting Fig. S2 for reversed-phase HPLC trace and Supporting Fig. S3 for NMR spectra of pure **4a**.

***N*-(14-(1-(3-(((2-(λ-azaneyl)-4-oxo-4,7-dihydro-3*H*-pyrrolo[2,3-*d*]pyrimidin-5-yl)methyl)amino)propyl)-1*H*-1,2,3-triazol-4-yl)-3,6,9,12-tetraoxatetradecyl)-6-((4*S*)-2-oxohexahydro-1*H*-thieno[3,4-*d*]imidazol-4-yl)hexanamide (4b).** To a solution of **2** (3.0 mg, 8.3 µmol, 1.3 eq) in a mixture of acetonitrile and water (1:1, v:v, 300 µL) was added a solution of N-(3,6,7,10,11,14-hexaoxaheptadec-16-yn-1-yl)-6-((4R,5S)-5-methyl-2-oxoimidazolidin-4-yl)hexanamide (106 µL, 6.37 µmol, 60 mM in DMSO, 1.0 eq) and copper sulfate (4.8 mg, 19 µmol, 3 eq) pre-dissolved in a minimal amount of water. After addition of ascorbic acid (22.4 mg, 127 µmol, 20 eq), the reaction was allowed to proceed for 3 h. The volatiles were removed in vacuo and the crude product re-dissolved in aqueous trifluoracetic acid (0.1%), containing acetonitrile (10%, v:v) and filtered. The filtrate was purified by reversed-phase chromatography (eluent A: aqueous trifluoroacetic acid (0.1%); eluent B: acetonitrile, linear gradient: 0-40% B in 400 mL, flow: 5.0 mL min^{-1}) to give **4b** (3.05 mg, 61 %) as an off-white solid. HR-ESI-

MS (m/z): $[M+H]^+$ found: 676.3893; $[M+H]^+$ calculated: 676.3889. See Supporting Fig. S2 for Reversed-Phase HPLC trace and Supporting Fig. S4 for NMR spectra of pure **4b**.

1.2. Homology analysis of preQ$_1$ candidates

Structural homology searches were performed on the 14 most enriched candidates using 51 riboswitch covariance models from the RFAM database (v.15.0)[2] with cmsearch and cmscan from INFERNAL v1.1.5[3]. Detected hits were then extended 100 nt upstream and searched for the occurrence of local stable hairpins upstream of the homologous regions using span distances S=(20,25,30) between two pairing candidates employing RNALfold from the ViennaRNA package v2.7.0 [4], as follows:

RNALfold -L <S> --noLP < <fasta_sequence> > <output>.

Among predicted structures, the gene with the genomic annotation CCL31 *RS08930/353-392* (corresponding to the *lmo2684* gene) consistently displayed a hairpin with the closest proximity to the homology-detected hit and the lowest free energy. Based on this refined region, pseudoknots were identified using pKiss program, with the subopt mode and pknotsRG strategy, as implemented in the RNA shapes studio[5] online server (https://bibiserv.cebitec.uni-bielefeld.de/pkiss?viewType=submission&subType=pkiss_function_subopt_p_1).

To detect conserved regions in the updated sequence relative to annotated preQ$_1$ sequences, we searched for homologs in the RNACentral database[6] and retrieved full sequences of all preQ$_1$ hit, using the RNACentral API. Multiple anchored-alignment was calculated using MLocARNA v2.0.1[7]:

mlocarna <fasta> --stockholm

The regions that form a predicted most stable hairpin loop were constrained and curated manually to match the structural annotation from the RFAM PreQ$_1$-I family (RF00522)(see Supporting Fig. S6b). To compare all hits with the current RFAM preQ$_1$ model, additional searches were done using cmsearch and cmalign to identify divergent structural regions of these sequences in relation to the canonical model. This confirmed the high conservation of our preQ$_1$ candidate sequence in the 3' tail region and the absence of sequence conservation in the canonical hairpin loop. We then searched in KEGG Sequence Similarity DataBase[8] for the *lmo2684* gene (ID: lmo:lmo2684, KO: K02761) and extracted 300 nt upstream of the annotated start codon for each of the 210 most similar genes. Regions were aligned using MAFFT v.7.525 (Katoh:2013), as follows:

mafft --localpair --maxiterate 1000 --thread 25 <fasta> > <alignment result>

To refine sequence selection in the alignment, we built a Hidden Markov Model (HMM) using HHMER (v3.4)[9] with the previously aligned RNACentral hits using hmmbuild:

hmmbuild <model.hmm> <alignment.sto>

and then searched this model against the extracted sequences, using hmmsearch:

hmmsearch -o <out_file> --tblout <tabular_file> model.hmm sequences.fasta

A multiple structural alignment of the 37 species with HMM hits was then calculated using MLocARNA (as above) without structural constraints. The resulting consensus secondary structure consisted of four sequence candidates that displayed variation at the nucleotide level in the sequence alignment (Fig. 5c). This alignment was visualized using R2R[10].

1.3. Sequence conservation analysis of upstream sequences from *lmo2684* gene and its orthologs

Using the previously calculated extended-region MAFFT alignment from KEGG Sequence Similarity Database, we identified the positions in the alignment of the detected *L. monocytogenes* preQ_1-like sequence, the adjacent upstream start codon (AUG_1), the amber stop codon (UAG), the Shine-Dalgarno sequences, and the canonical start codon (AUG) of the *lmo2684* gene. We selected species with previously detected pre-Q_1-like candidates using HMM model (as described in Section 1.2), comprising 35 *L. monocytogenes* strains, one *L. innocua*, and one *L. welshimeri*. The corresponding sequences were extracted from the alignment, and the alignment was restricted to the start and end positions covering all previously mentioned elements. Sequence conservation and ORF protein products were then accessed using Jalview v.2.11.4.1 [11], as shown in Supporting Fig. S11 c, d.

2. Supporting Figures

a

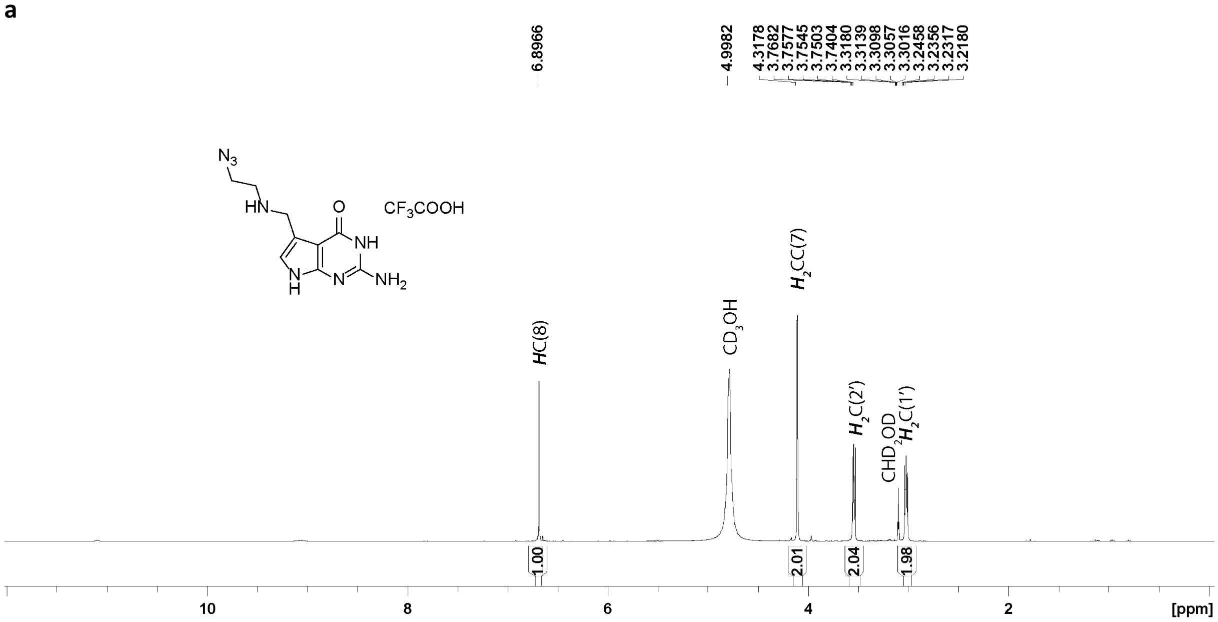

b

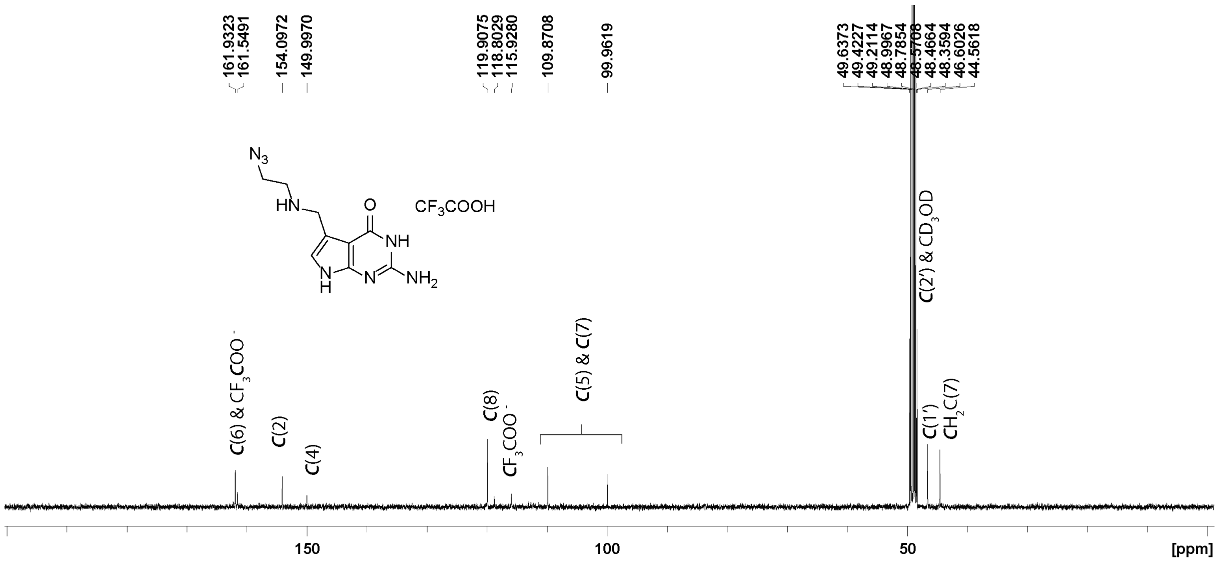

Supporting Figure S1. NMR spectroscopic characterization of compound **2**. **a,** ^{1}H-NMR (400 MHz, CD_3OD, 25 °C) spectrum. **b,** ^{13}C-NMR (100 MHz, CD_3OD, 25 °C) spectrum

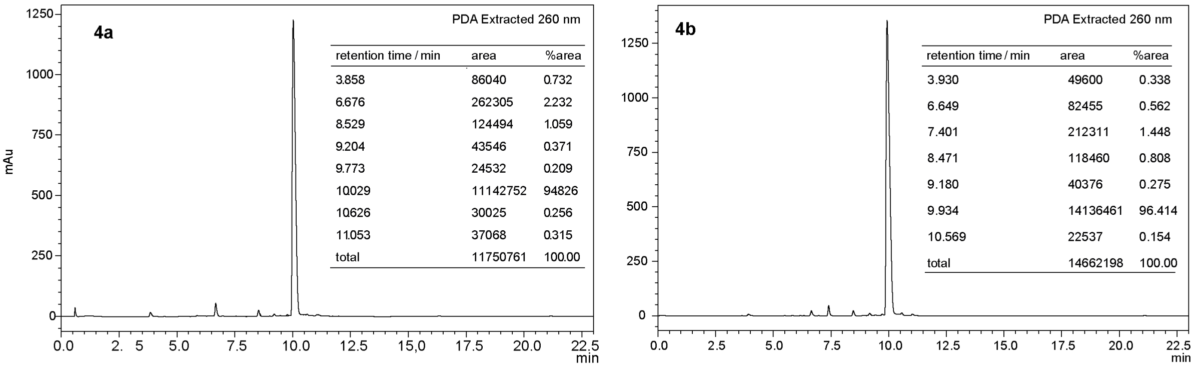

Supporting Fig. S2. RP-HPLC analysis of purified compounds **4a** und **4b** indicating ≥ 95% purity. Analysis was performed at 25 °C, linear gradient: 0-50% ACN in 0.1% formic acid.

a

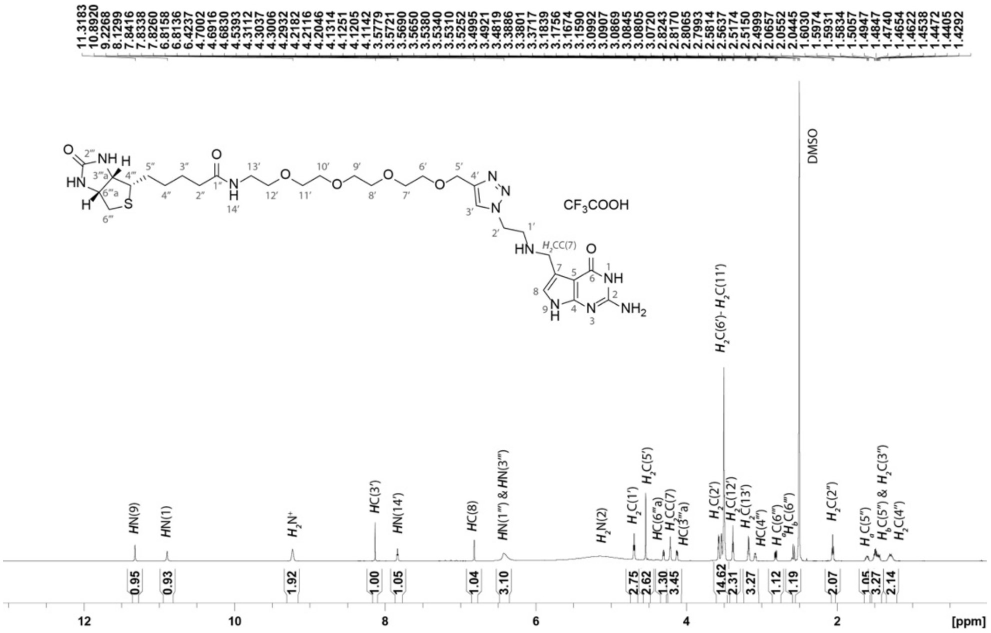

^{1}H NMR (DMSO-d_6, 700 MHz, 25 °C) of 4a.

b

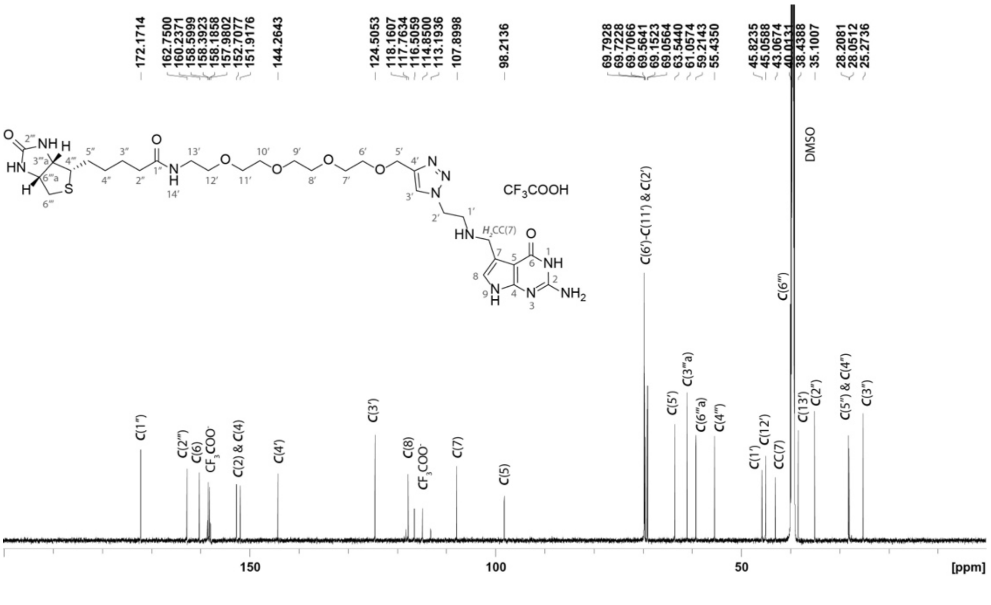

^{13}C NMR (DMSO-d_6, 176 MHz, 25 °C) of 4a.

Supporting Fig. S3. NMR spectra of 4a.

a. ^{1}H NMR (DMSO-d_6, 700 MHz, 25 °C): δ 11.32 (s, 1H, *H*N(9)), 10.89 (s, 1H, *H*N(1)), 9.23 (bs, 2H, H_2N^+)), 8.13 (s, 1H, *H*C(3')), 7.83 (t, J_{HH} = 5.5 Hz, 1H, *H*N(14')), 6.81 (d, J_{HH} = 1.5 Hz, 1H, *H*C(8)), 6.42 (bs, 2H, *H*N(1''') & *H*N(3''')), 5.15 (bs, 2H, H_2N(2)), 4.69 (t, J_{HH} = 6.0 Hz, 2H, H_2C(1')), 4.54 (s, 2H, H_2C(5')), 4.30 (dd, J_{HH} = 5.3, 7.4 Hz, 1H, *H*C(6'''a)), 4.21 (t, J_{HH} = 4.8 Hz, 2H, H_2CC(7)), 4.12 (dd, J_{HH} = 4.46, 7.6 Hz, 1H, *H*C(3'''a)), 3.59-3.52 (m, 2H, H_2C(2')), 3.50 (s, 12H, H_2C(6')-H_2C(11')), 3.38 (t, J_{HH} = 5.9 Hz, 2H, H_2C(12')), 3.17 (q, J_{HH} = 5.8 Hz, 2H, H_2C(13')), 3.11-3.06 (m, 1H, *H*C(4''')), 2.81 (dd, J_{HH} = 5.1, 12.5 Hz, 1H, H_aC(6''')), 2.57 (d, J_{HH} = 12.5 Hz, 1H, H_bC(6''')), 2.06 (t, J_{HH} = 7.5 Hz, 2H, H_2C(2'')), 1.64-1.56 (m, 1H, H_aC(5'')), 1.55-1.40 (m, 3H, H_2C(3'')) & H_bC(5'')), 1.35-1.21 (m, 2H, H_2C(4'')) ppm.

b. ^{13}C NMR (DMSO-d_6, 176 MHz, 25 °C): δ 172.2 *C*(1''), 162.8 *C*(2'''), 160.2 *C*(6), 158.3 (q, J_{CF} = 36.0 Hz, CF_3*C*OO$^-$), 152.7, 151.9 *C*(2) & *C*(4), 144.3 *C*(4'), 124.5 C(3'), 118.2 *C*(8), 115.8 (q, J_{CF} = 290.4 Hz, *C*F$_3$COO$^-$), 107.9 *C*(7), 98.2 *C*(5), 69.8, 69.7, 69.7, 69.6, 69.2, 69.1 *C*(6')-*C*(11') & *C*(2'), 63.5 *C*(5'), 61.1 *C*(3'''a), 59.2 *C*(6'''a), 55.4 *C*(4'''), 45.8 *C*(1'), 45.1 *C*(12'), 43.1 *C*H$_2$C(7), 40.0 *C*(6'''), 38.4 *C*(13'), 35.1 *C*(2''), 28.2, 28.1 *C*(5'') & *C*(4''), 25.3 *C*(3'') ppm.

a

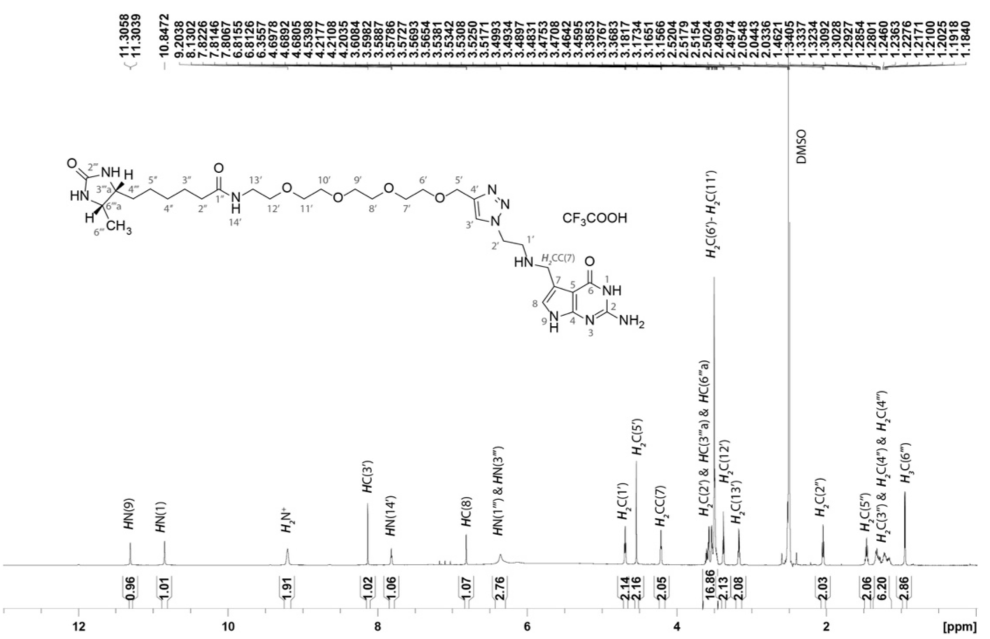

^{1}**H NMR (DMSO-*d$_6$*, 700 MHz, 25 °C) of 4b.**

b

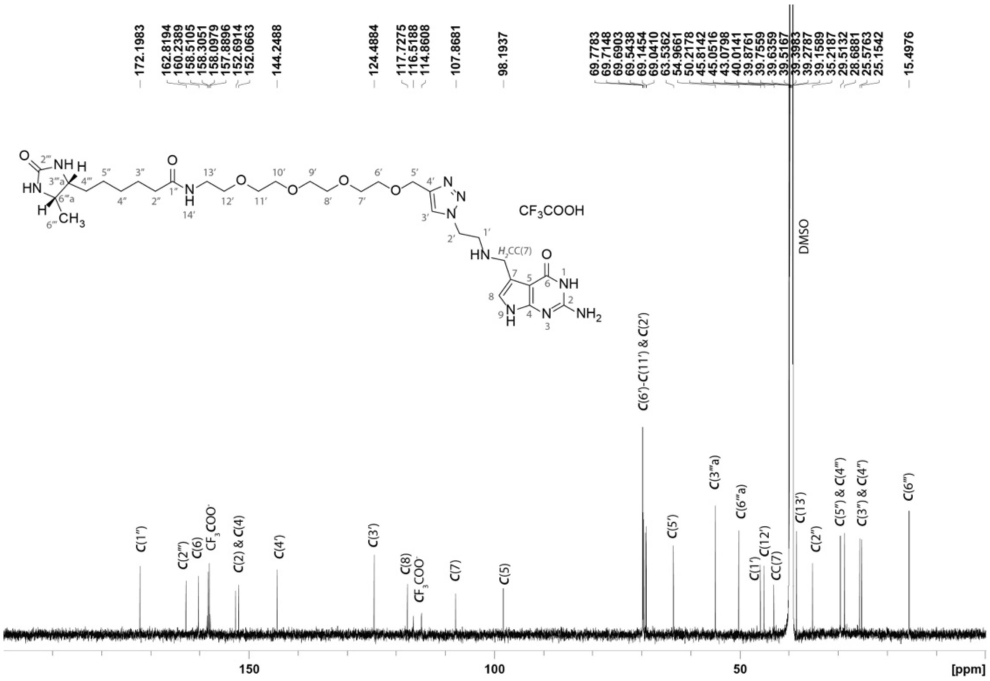

^{13}**C NMR (DMSO-*d$_6$*, 176 MHz, 25 °C) of 4b**.

Supporting Fig. S4. NMR spectra of 4b.

a. ^{1}**H NMR (DMSO-*d$_6$*, 700 MHz, 25 °C):** δ 11.30 (d, J_{HH} = 1.5 Hz, 1H, *H*N(9)), 10.85 (s, 1H, *H*N(1)), 9.20 (bs, 2H, H_2N^+)), 8.13 (s, 1H, *H*C(3')), 7.81 (t, J_{HH} = 5.5 Hz, 1H, *H*N(14')), 6.81 (d, J_{HH} = 2.0 Hz, 1H, *H*C(8)), 6.36 (bs, 2H, *H*N(1''') & *H*N(3''')), 5.64 (bs, 2H, H_2N(2)), 4.69 (t, J_{HH} = 6.0 Hz, 2H, H_2C(1')), 4.54 (s, 2H, H_2C(5')), 4.21 (t, J_{HH} = 4.8 Hz, 2H, H_2CC(7)), 3.62-3.45 (m, 16H, H_2C(2') & *H*C(3'''a) & *H*C(6'''a) & *H*C(6')-*H*C(11')), 3.38 (t, J_{HH} = 6.0 Hz, 2H, H_2C(12')), 3.17 (q, J_{HH} = 5.8 Hz, 2H, H_2C(13')), 2.04 (t, J_{HH} = 7.5 Hz, 2H, H_2C(2'')), 1.50-1.43 (m, 2H, H_2C(5'')), 1.38-1.13 (m, 6H, H_2C(3'') & H_2C(4'') & H_2C(4''')), 0.95 (d, J_{HH} = 6.4 Hz, 3H, H_3C(6''') ppm.

b. ^{13}**C NMR (DMSO-*d$_6$*, 176 MHz, 25 °C):** δ 172.2 *C*(1''), 162.8 *C*(2'''), 160.2 *C*(6), 158.2 (q, J_{CF} = 36.3 Hz, CF_3COO^-), 152.7, 152.1 *C*(2) & *C*(4), 144.2 *C*(4'), 124. C(3'), 117.7 *C*(8), 115.8 (q, J_{CF} = 289.8 Hz, CF_3COO^-), 107.9 *C*(7), 98.2 *C*(5), 69.8, 69.7, 69.7, 69.5, 69.1, 69.0 *C*(6')-*C*(11') & *C*(2'), 63.5 *C*(5'), 54.9 C(3'''a) 50.2 C(6'''a) 45.8 *C*(1'), 45.1 *C*(12'), 43.1 CH_2C(7), 38.4 *C*(13'), 35.2 *C*(2''), 29.5, 28.7 *C*(5'') & *C*(4'''), 25.6*, 25.2* C(3'') & *C*(4''), 15.5 C(6''') ppm.

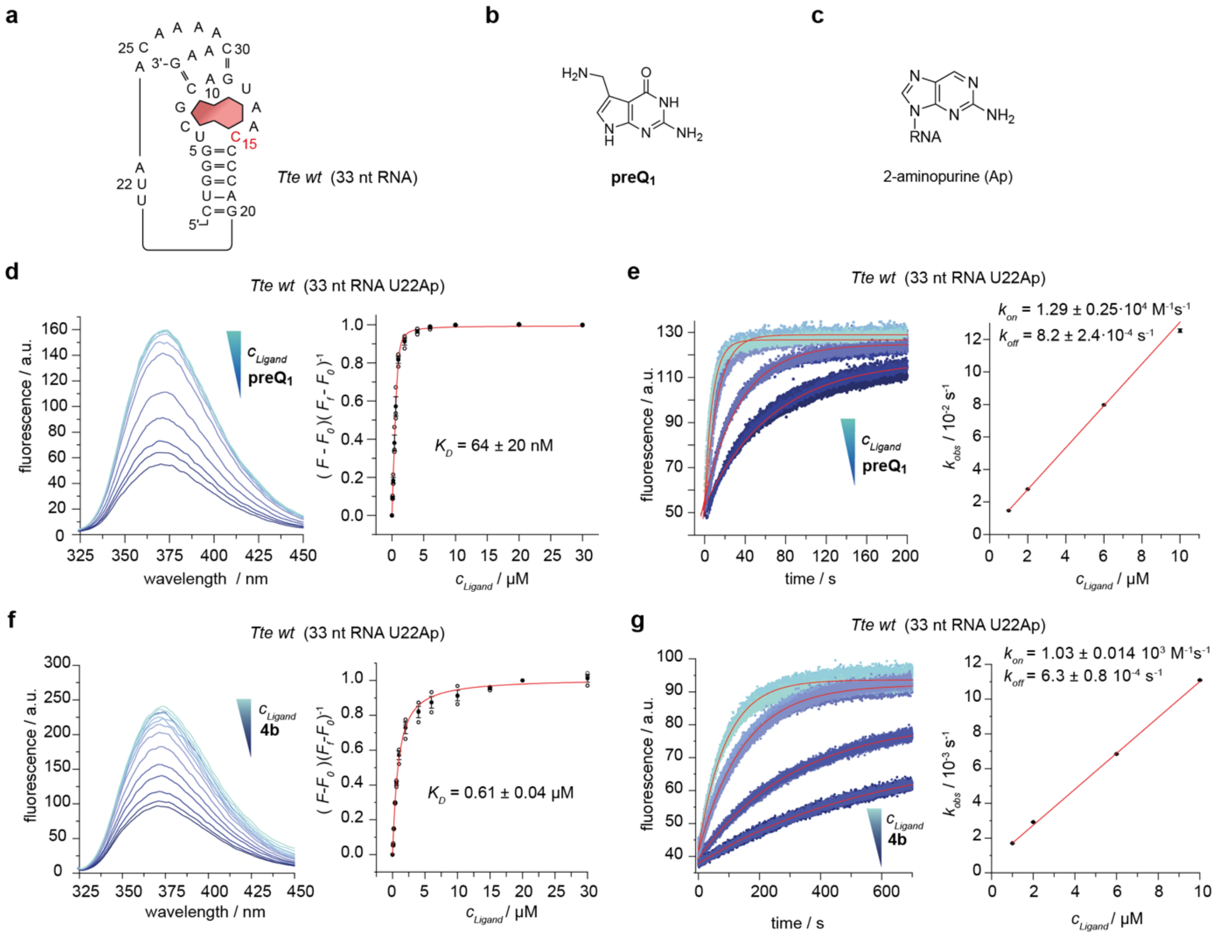

Supporting Fig. S5. Binding of $preQ_1$ to the $preQ_1$-I riboswitch from *Thermoanaerobacter tengcongensis.* **a,** Secondary structure of the *Tte* $preQ_1$-I riboswitch. Nucleotide C15 (red) pairs to $preQ_1$ in Watson-Crick mode, the ligand is represented by the russet nonagon. **b,** Chemical structure of $preQ_1$. **c,** Chemical structure of the fluorescent nucleobase 2-aminopurine, that was used as structural probe. **d,** Affinity (K_D) determination of $preQ_1$ and *Tte* U22Ap RNA using a 2-aminopurine fluorescence assay. Experiments were performed as 3 independent replicas, open circles indicate individual data points, filled circles mean values (reported ± s.e.m). The fluorescence change upon ligand addition of a representative experiment is shown. **e,** Kinetics (k_{on} and k_{off}) determination of $preQ_1$ and *Tte* U22Ap RNA. Open circles indicate individual data points, filled circles mean values (reported ± s.e.m). The fluorescent time traces are shown. **f,** same as **d** but for ligand **4b**. **g,** same as **e** but for ligand **4b**.

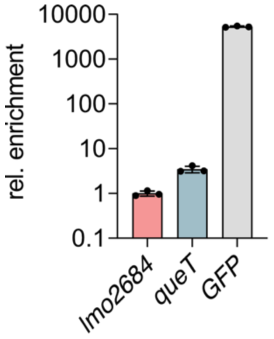

Supporting Fig. S6. RT-qPCR analysis of total RNA extracted from *L. monocytogenes* (*lmo2684*, *queT*) or *E. coli* (*GFP*). Before cDNA synthesis, equal amounts of RNA from both bacteria were supplemented with equal amounts of *in vitro* transcribed *tetR* RNA. Ct values of the indicated genes were normalized to Ct values of *tetR* and further normalized to *lmo2684* to express relative enrichment compared to *lmo2684.* Mean ± SD of three technical replicates is shown**.**

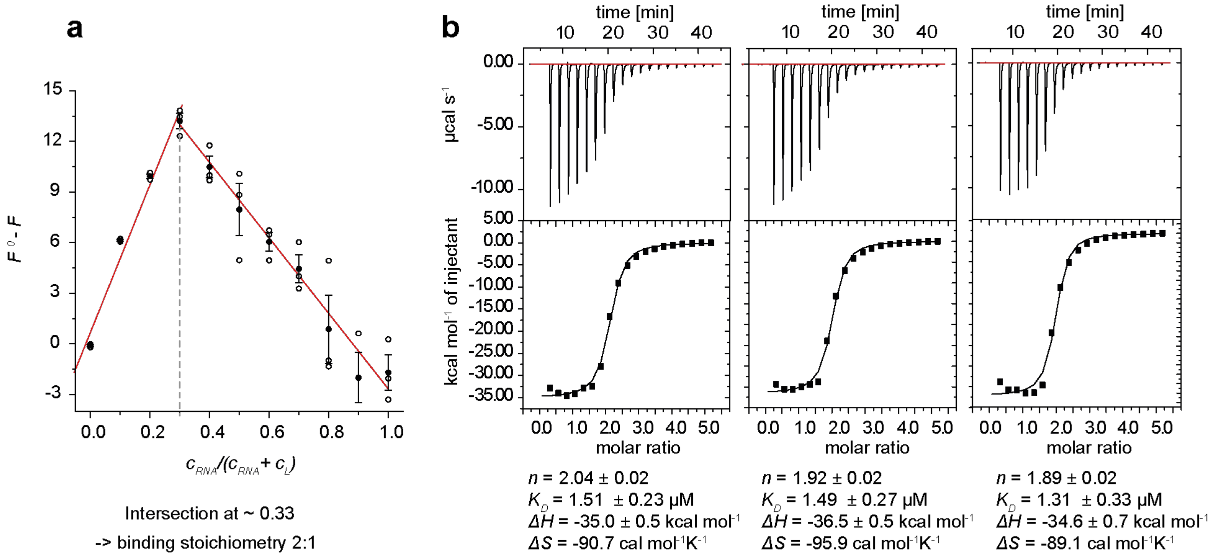

Supporting Fig. S7. Evaluation of the binding stoichiometry of *queT* RNA and $preQ_1$. **a,** Job plot analysis based on 2ApFold experiments employing *queT* A29Ap and $preQ_1$. A maximum of F_0-F at around 0.33 is indicative for 2:1 binding stoichiometry. Experiments were conducted as 3 independent replicates. Open circles represent individual data points; filled circles mean values (reported ± s.e.m). Red lines are a linear fit to the slope of either side of the maximum. **b,** Isothermal titration calorimetry of *queT* RNA and $preQ_1$ at 25 °C. Thermograms were analyzed by a "set of identical sites" model supporting a 2:1 binding stoichiometry (n = 2). Experiments were conducted as 3 independent replicates.

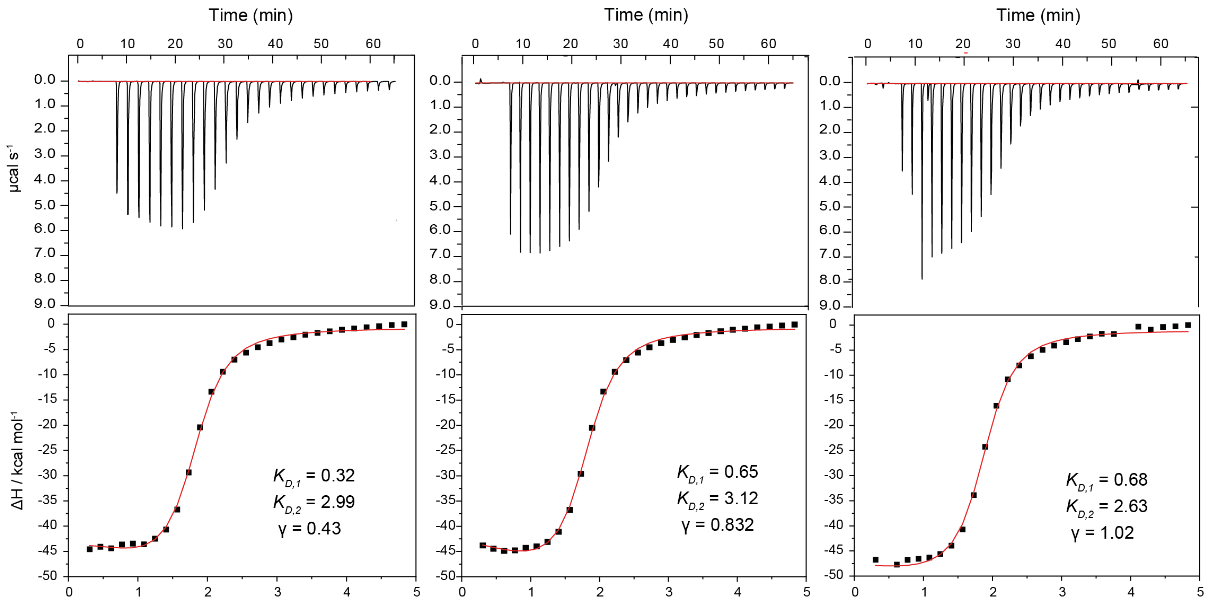


```
# Parameter Fit_value Confidence_lower Confidence_upper
# Offset_1_(kcal_mol^-1)    -0.67416140    -1.18374323    -0.16457958
# Eta                        0.89286412     0.82953714     0.95619110
# DeltaH_A1_(kcal_mol^-1)  -44.88292742   -45.54644404   -44.21941079
# DeltaH_B1_(kcal_mol^-1)  -40.40054357   -44.10387402   -36.69721313
# DeltaH_A2_(kcal_mol^-1)  -51.93059031   -55.48288511   -48.37829551
# DeltaH_B2_(kcal_mol^-1)  -47.44820647   -52.62253821   -42.27387472
# DeltaG_A1_(kcal_mol^-1)   -0.42085245    -3.36727888     2.52557398
# DeltaG_B1_(kcal_mol^-1)   -0.05974933    -2.83338256     2.71388391
# DeltaG_A2_(kcal_mol^-1)    0.04125231    -0.85838060     0.94088521
# DeltaG_B2_(kcal_mol^-1)    0.40235543    -0.02078521     0.82549607
# KD_A1_(uM)                 0.50518290     0.00423902    60.20488765
# KD_B1_(uM)                 0.90760727     0.01008028    81.71904917
# KD_A2_(uM)                 1.06922280     0.24839729     4.60245515
# KD_B2_(uM)                 1.92095651     0.96683824     3.81664043
# Alpha                      0.47247674     0.00498129    44.81450713
# KD_1_(uM)                  0.32454053     0.00320404    32.87308369
# KD_2_(uM)                  2.99017930     1.50982727     5.92198354
# Gamma                      0.43414190     0.00434356    43.39280743
```

```
# Parameter Fit_value Confidence_lower Confidence_upper
# Offset_1_(kcal_mol^-1)    -0.60726897    -1.05830543    -0.15623251
# Eta                        0.87677733     0.82728050     0.92627415
# DeltaH_A1_(kcal_mol^-1)  -46.22783504   -48.51597911   -43.93969096
# DeltaH_B1_(kcal_mol^-1)  -37.19437084   -37.65930187   -36.72943982
# DeltaH_A2_(kcal_mol^-1)  -57.86598574   -61.70243040   -54.02954109
# DeltaH_B2_(kcal_mol^-1)  -48.83252155   -51.87662132   -45.78842177
# DeltaG_A1_(kcal_mol^-1)    0.00647274    -2.05525444     2.06819992
# DeltaG_B1_(kcal_mol^-1)    0.37150062    -1.80148098     2.54448222
# DeltaG_A2_(kcal_mol^-1)    0.06590504    -0.52037773     0.65218780
# DeltaG_B2_(kcal_mol^-1)    0.43093292     0.07399794     0.78786790
# KD_A1_(uM)                 1.01055739     0.03562676    28.66458444
# KD_B1_(uM)                 1.82715698     0.05377698    62.08051927
# KD_A2_(uM)                 1.11285766     0.42985176     2.88111458
# KD_B2_(uM)                 2.01212288     1.12756669     3.59059783
# Alpha                      0.90807425     0.02803754    29.41052856
# KD_1_(uM)                  0.65068106     0.02240830    18.89415197
# KD_2_(uM)                  3.12498054     1.88823015     5.17177602
# Gamma                      0.83287694     0.02732329    25.38801087
```

```
Parameter Fit_value Confidence_lower Confidence_upper
# Offset_1_(kcal_mol^-1)    -0.99315432    -1.82157842    -0.16473022
# Eta                        0.90918793     0.71817421     1.10020165
# DeltaH_A1_(kcal_mol^-1)  -34.59139541   -35.53319518   -33.64959565
# DeltaH_B1_(kcal_mol^-1)  -60.53220508   -69.93329247   -51.13111770
# DeltaH_A2_(kcal_mol^-1)  -37.93058596   -48.23051236   -27.63065957
# DeltaH_B2_(kcal_mol^-1)  -63.87139563   -77.84837365   -49.89441761
# DeltaG_A1_(kcal_mol^-1)    0.17748388    -7.50674700     7.86171476
# DeltaG_B1_(kcal_mol^-1)    0.19262319    -7.47994400     7.86519037
# DeltaG_A2_(kcal_mol^-1)    0.16279068    -0.14048216     0.46606352
# DeltaG_B2_(kcal_mol^-1)    0.17792999    -0.11726933     0.47312931
# KD_A1_(uM)                 1.33371500     0.00000513 346508.48665843
# KD_B1_(uM)                 1.36688150     0.00000536 348468.03362944
# KD_A2_(uM)                 1.30229554     0.79617773     2.13014459
# KD_B2_(uM)                 1.33468071     0.82673593     2.15470567
# Alpha                      1.02412621     0.00000398 263503.05149223
# KD_1_(uM)                  0.67504729     0.00000264 172931.03395390
# KD_2_(uM)                  2.63697625     1.87082763     3.71688104
# Gamma                      1.02397175     0.00000398 263523.64836165
```

Supporting Fig. S8. Three independent replicates for affinity (K_D) determination of $preQ_1$ with 41 nt *queT* RNA using isothermal titration calorimetry (ITC).

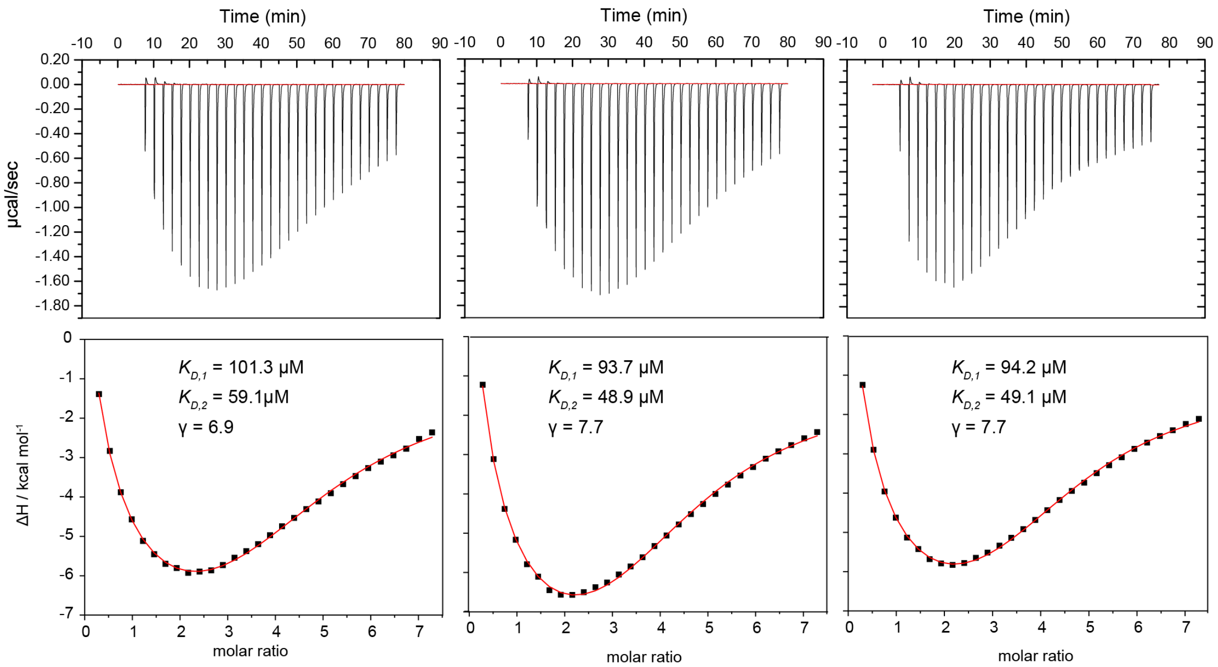

Parameter	value	confidence upper	confidence lower
# Offset_1_(kcal_mol^-1)	-1.05429214	-1.47100473	-0.63757955
# Eta	1.46590043	1.37136600	1.56043485
# DeltaH_A1_(kcal_mol^-1)	-5.98157977	-9.88148825	-2.08167130
# DeltaH_B1_(kcal_mol^-1)	18.32108331	12.40369508	24.23847155
# DeltaH_A2_(kcal_mol^-1)	-40.13881306	-49.83998341	-30.43764271
# DeltaH_B2_(kcal_mol^-1)	-15.83614997	-22.46094706	-9.21135288
# DeltaG_A1_(kcal_mol^-1)	3.02655665	3.00516388	3.04794942
# DeltaG_B1_(kcal_mol^-1)	3.30159286	2.88512656	3.71805916
# DeltaG_A2_(kcal_mol^-1)	1.85424411	1.55717264	2.15131558
# DeltaG_B2_(kcal_mol^-1)	2.12928032	1.83818744	2.42037321
# KD_A1_(uM)	165.01168985	159.16242077	171.07592141
# KD_B1_(uM)	262.43986478	129.98430406	529.86922632
# KD_A2_(uM)	22.83379534	13.83305459	37.69103968
# KD_B2_(uM)	36.31559780	22.22355900	59.34344916
# Alpha	7.22664311	4.37233026	11.94428774
# KD_1_(uM)	101.31123657	77.08660355	133.14851325
# KD_2_(uM)	59.14939314	41.33941734	84.63231787
# Gamma	6.85121055	4.57222298	10.26614104

#Parameter	value	confidence upper	confidence lower
Offset_1_(kcal_mol^-1)	-1.22717477	-1.55759361	-0.89675594
# Eta	1.37938980	1.30378419	1.45499541
# DeltaH_A1_(kcal_mol^-1)	-5.40568200	-12.44072430	1.62936030
# DeltaH_B1_(kcal_mol^-1)	33.08306145	27.34793556	38.81818734
# DeltaH_A2_(kcal_mol^-1)	-56.15463528	-68.68697572	-43.62229484
# DeltaH_B2_(kcal_mol^-1)	-17.66589184	-26.30742340	-9.02436027
# DeltaG_A1_(kcal_mol^-1)	2.88051602	2.84176825	2.91926379
# DeltaG_B1_(kcal_mol^-1)	3.45493830	3.01975772	3.89011889
# DeltaG_A2_(kcal_mol^-1)	1.53944392	1.16288527	1.91600256
# DeltaG_B2_(kcal_mol^-1)	2.11386620	1.89919357	2.32853883
# KD_A1_(uM)	129.27815037	121.09370846	138.01575966
# KD_B1_(uM)	340.87721998	163.52888041	710.56120982
# KD_A2_(uM)	13.44217407	7.11931251	25.38054670
# KD_B2_(uM)	35.44397035	24.67057988	50.92199048
# Alpha	9.61735428	5.07652638	18.21984100
# KD_1_(uM)	93.73066710	75.86001428	115.81118244
# KD_2_(uM)	48.88614442	35.65765351	67.02222049
# Gamma	7.66930329	5.18628081	11.34111615

Parameter	value	confidence upper	confidence lower
# Offset_1_(kcal_mol^-1)	-1.03526700	-1.27475222	-0.79578178
# Eta	1.38653077	1.32494423	1.44811732
# DeltaH_A1_(kcal_mol^-1)	-5.21284153	-10.19461774	-0.23106531
# DeltaH_B1_(kcal_mol^-1)	27.45323984	23.25654262	31.64993706
# DeltaH_A2_(kcal_mol^-1)	-47.98357202	-56.90178208	-39.06536196
# DeltaH_B2_(kcal_mol^-1)	-15.31749065	-21.40880487	-9.22617643
# DeltaG_A1_(kcal_mol^-1)	2.89090809	2.86404481	2.91777138
# DeltaG_B1_(kcal_mol^-1)	3.44339645	3.08277741	3.80401549
# DeltaG_A2_(kcal_mol^-1)	1.55910599	1.24654944	1.87166253
# DeltaG_B2_(kcal_mol^-1)	2.11159434	1.93373804	2.28945064
# KD_A1_(uM)	131.25835470	125.44247460	137.34387602
# KD_B1_(uM)	333.37057311	181.42906202	612.55863742
# KD_A2_(uM)	13.87824742	8.19084904	23.51474805
# KD_B2_(uM)	35.24803665	26.11091969	47.58254792
# Alpha	9.45784801	5.57111808	16.05618254
# KD_1_(uM)	94.17767666	78.87773727	112.44535009
# KD_2_(uM)	49.12628407	37.81071249	63.82825467
# Gamma	7.66821089	5.52164083	10.64927259

Supporting Fig. S9. Three independent replicates for affinity (K_D) determination of preQ$_1$-biotin conjugate **4a** with 41 nt *queT* RNA using isothermal titration calorimetry (ITC).

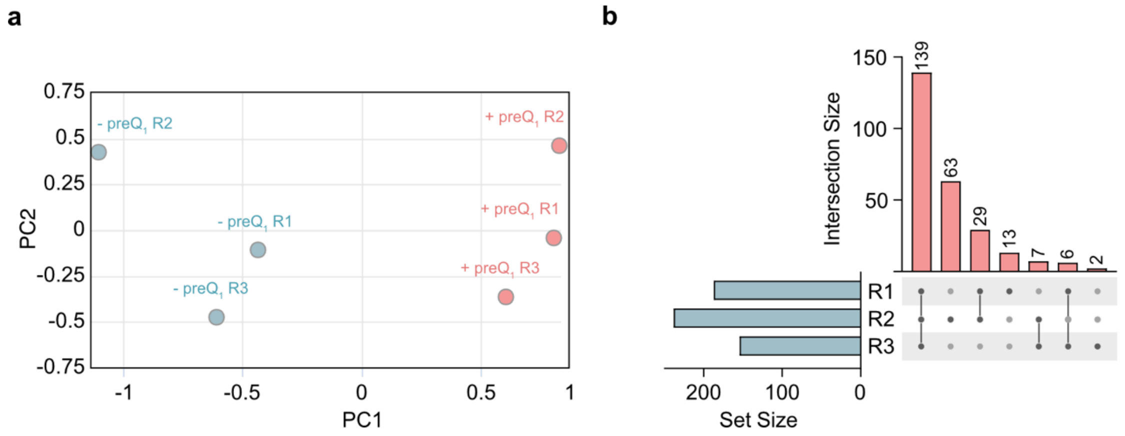

Supporting Fig. S10. DTB-preQ_1 pull-down of total RNA from *L. monocytogenes*. **a**, PCA plot of three replicates each of DTB-preQ_1 (red) or mock (blue) pull-down samples shows segregation of the two conditions. **b**, Illustration of the number and distribution of identified enriched peaks in the three replicates.

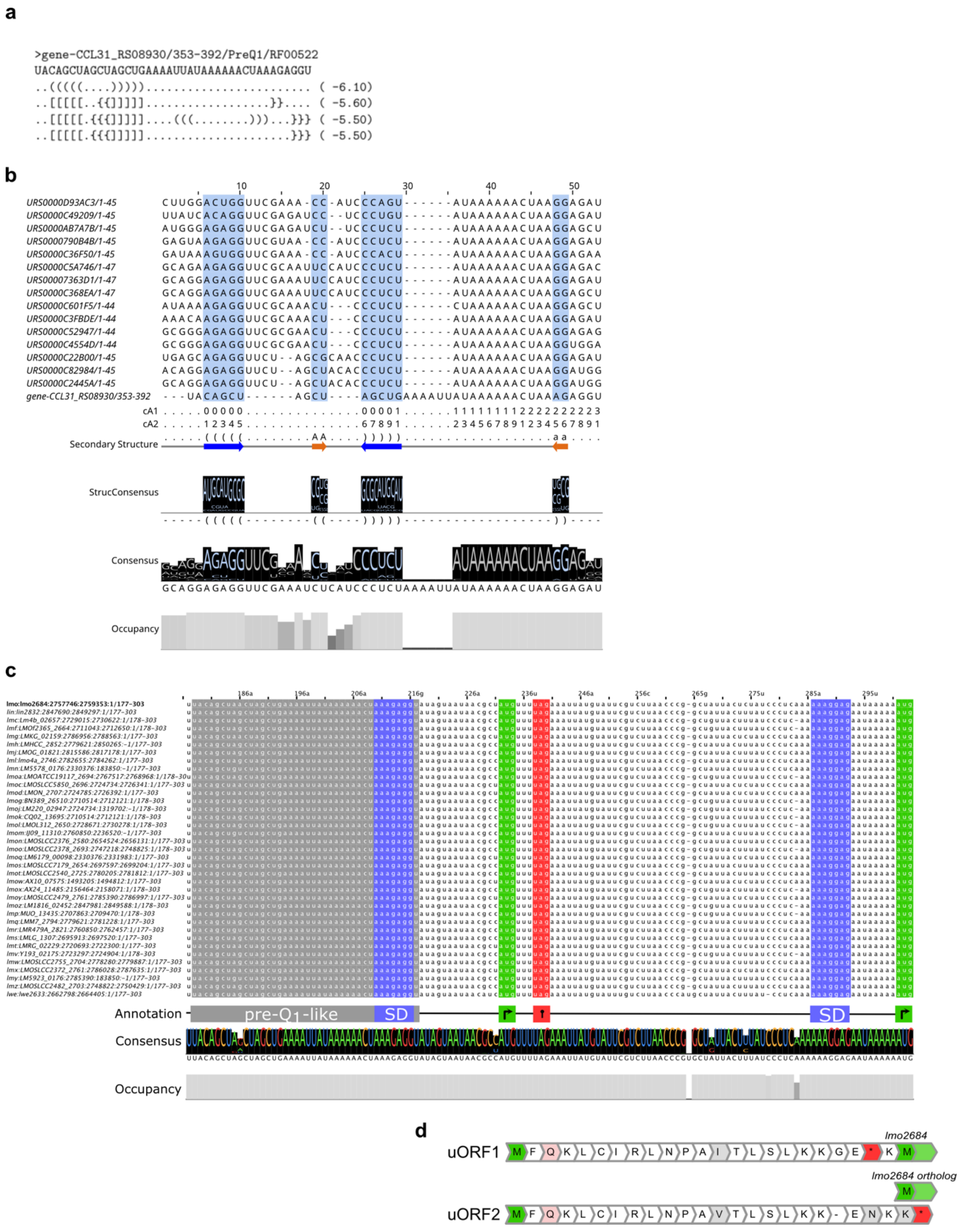

Supporting Fig. S11. Sequence characterization of preQ$_1$ candidate sequence upstream of *lmo2684*. **a,** Suboptimal structure prediction of preQ$_1$ -like sequence associated with *lmo2684* calculated using pKiss. Folding free energies are shown in parentheses. **b,** Multiple structural alignment with annotated preQ$_1$ sequences from RNACentral. RNA helices are highlighted in blue, the position of the calculated pseudoknot is indicated with opposing orange arrows. **c,** Sequence alignment of genomic upstream regions from *lmo2684* orthologs, retrieved from KEGG SSDB database (https://www.kegg.jp/ssdb-bin/ssdb_gclust?org_gene=lmo:lmo2684) which have pre-Q$_1$-like sequences (gray box). Start and stop codons are shown in green and red, respectively. Blue boxes indicate predicted Shine-Dalgarno (SD) sequences. **d,** Depending on the *Listeria* strain, two highly similar peptides could be translated from the upstream ORF by preQ$_1$-mediated stop codon suppression. A Q (highlighted in pale red) was introduced into the sequence as a place holder instead of the stop.

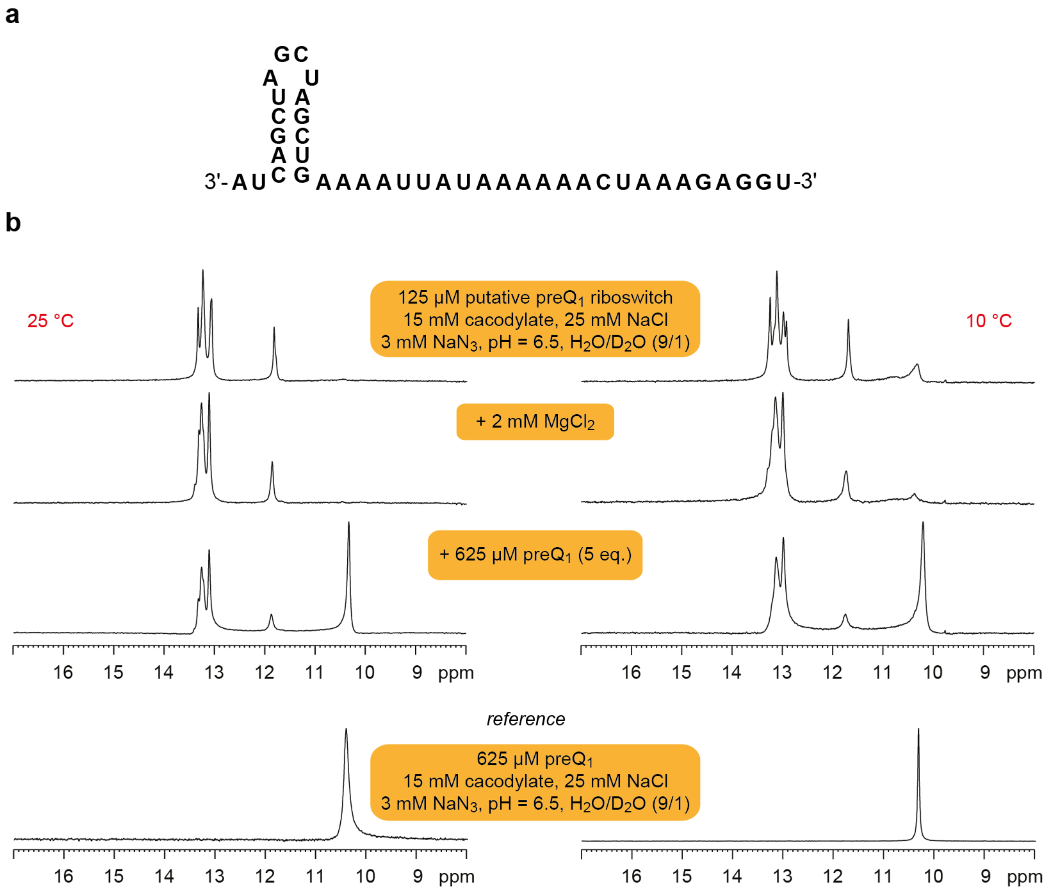

Supporting Fig. S12. No binding was detected by NMR spectroscopy between $preQ_1$ and a conserved region upstream of *lmo2684* partially resembling a $preQ_1$ riboswitch motif. **a,** Secondary structure of the 40 nt RNA *lmo2684* upstream region used for NMR experiments. Note that the hairpin substructure contains a palindromic sequence, which can lead to the formation of a competing duplex. **b,** ^{1}H-NMR spectroscopy of the candidate RNA in the absence or presence of $preQ_1$ at two different temperatures (10 °C, 25 °C). No changes in the imino proton chemical shift region are observed as would be expected if a structural rigidification of a high-affinity RNA-ligand complex is formed. *reference*, $preQ_1$ only.

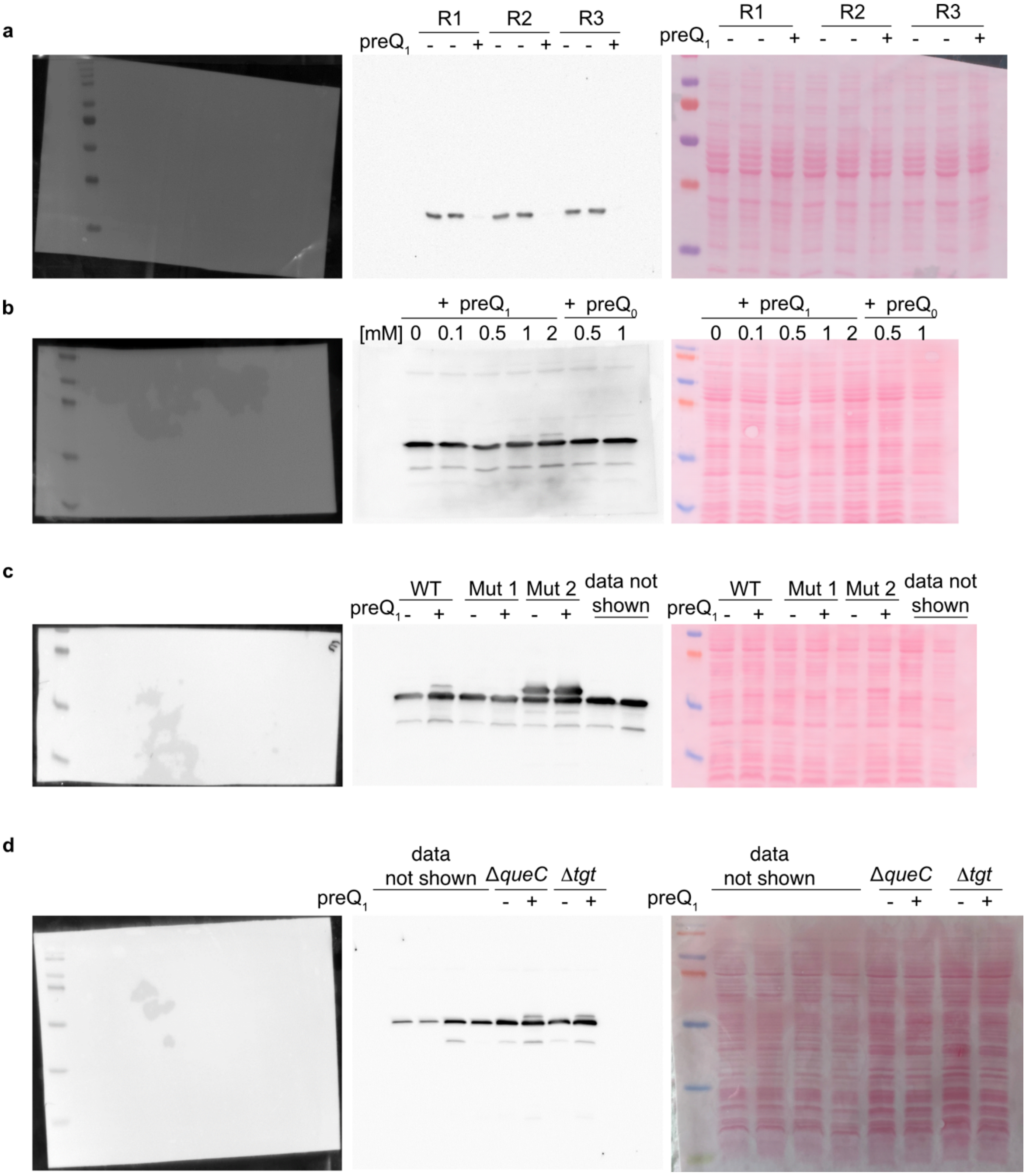

Supporting Fig. S13. Uncropped images of Western blot membranes shown in Figures 3 and 5. *Left images*, images of membranes; *middle images*, Chemiluminescence recordings; *right panels*, images of Ponceau-stained membranes. **a,** Raw images corresponding to Fig. 3a, only one experiment (R1) of three (R2, R3) is shown in Fig. 3a. **b-d**, Raw images corresponding to Fig. 5e (**b**), 5f (**c**) and 5g (**d**). “data not shown” designates lanes that were not shown in the final figures.

3. Supporting Tables

Supporting Table S1: Top 14 enriched sequences of DTB-preQ$_1$ (**4b**) transcriptome pull-down.

#	chr	gene ID	gene name	**start**	end	R1_plus.fc[a]	R2_plus.fc[a]	R3_plus.fc[a]	-log$_{10}$ R1_plus.qval	-log$_{10}$ R2_plus.qval	-log$_{10}$ R3_plus.qval
1	NZ_MWLS01000006.1	*lmo2434*	*gadD3*	59205	60960	14.83	5.77	10.84	1202.83	179.96	227.61
2	NZ_MWLS01000004.1	*lmo0355*		115487	117714	5.38	5.07	5.69	353.05	143.92	79.79
3	NZ_MWLS01000005.1	*lmo1906*	*mgsA*	124927	129853	4.57	5.45	4.06	91.53	154.52	18.54
4	NZ_MWLS01000002.1	*lmo2684*		470614	472244	6.28	2.61	4.48	153.23	25.56	21.41
5	NZ_MWLS01000001.1	*lmo0554*		1161287	1163920	3.76	4.15	4.09	438.48	209.25	134.11
6	NZ_MWLS01000001.1	*lmo1666*	*lapB*	19912	27731	3.95	3.63	4.39	146.08	96.32	40.12
7	NZ_MWLS01000001.1	*lmo1238*	*rph*	518941	522230	3.17	4.51	3.08	41.00	108.67	9.19
8	NZ_MWLS01000001.1	*lmo1454*	*rpoD*	254431	258895	3.08	3.25	3.69	161.28	158.86	46.32
9	NZ_MWLS01000002.1	*lmo2770*	*gshAB*	370130	372418	2.89	3.35	3.72	40.57	85.48	15.91
10	NZ_MWLS01000005.1	*lmo1809*	*plsX*	27086	35940	5.47;1.62	3.22	6.04;1.94	145.11;4.35	106.78	43.01;2.05
11	NZ_MWLS01000002.1	*lmo0202*	*hly*	29904	31647	2.94	2.89	3.68	157.88	98.93	53.65
12	NZ_MWLS01000001.1	*lmo1644*		51922	58826	1.47;3.45	3.15	1.76;3.42	2.15;54.53	56.88	1.24;12.67
13	NZ_MWLS01000001.1	*lmo0788*		945967	950505	2.96	3.45	2.81	64.16	56.06	11.08
14	NZ_MWLS01000003.1	*lmo2362*	*gadD2*	292567	295529	2.37	2.93	3.24	50.58	36.82	23.36

[a] Cells can contain two values when algorithm identified two peaks.

Supporting Table S2: Sequences of synthetic RNAs and molecular weight (m.w.) determined by electrospray ionization (ESI) mass spectrometric measurements in the negative mode.

#	Short name	Sequence (5‘ to 3‘)[a]	nt	m.w. (calculated)	m.w. (found)
1	*Tte*	CUG GGU CGC AGU AAC CCC AGU UAA CAA AAC AAG	33	10582.50	10582.49
2	*Tte* U22Ap	CUG GGU CGC AGU AAC CCC AGU ApAA CAA AAC AAG	33	10605.75	10605.54
3	*queT*	ACG UGG UUC AUU CAU ACC AUC CCA CGU AAA AAA ACU AGG AG	41	13099.99	13099.34
4	*queT* A29Ap	ACG UGG UUC AUU CAU ACC AUC CCA CGU AApA AAA ACU AGG AG	41	13099.99	13099.38

[a] 2-aminopurine (Ap) modifications are highlighted in red.

Supporting Table S3: Sequences of DNA primers used for qPCR and cloning.

#	Short name	Sequence (5‘ to 3‘)	Usage
1	*q yhhQ fw*	CGTAGCCCGGATGCCTTTAT	qPCR
2	*q yhhQ rev*	ACGCCATACATTGGCAGGAA	qPCR
3	*q BglA fw*	ACTGACGGTCCAACACCAGA	qPCR
4	*q BglA rev*	CGGGACCAAGCGATGGAAGT	qPCR
5	*q queT fw*	TTTGGCGTAGCACAATCGGC	qPCR
6	*q queT rev*	TCCCAAGCGATAATTGCCATCG	qPCR
7	*q Tet-R fw*	CTCGCCCAGAAGCTAGGTGTAGAGC	qPCR
8	*q Tet-R rev*	GCCTATCTAACATCTCAATGGCTAAGGCG	qPCR
9	*q BglA i.v.Tr fw*	TGCAAACCAATTCGAAGGCG	qPCR
10	*q BglA i.v.Tr rev*	CTGGTGTTGGACCGTCAGTA	qPCR
11	*q queT i.v.Tr fw*	ATTGCCACACGTGGTTCATTC	qPCR
12	*q queT i.v.Tr rev*	ACTACATACAGTGCGGCGAT	qPCR
13	*q GFP fw*	ACGACGGCAACTACAAGACC	qPCR
14	*q GFP rev*	CCTCCTTGAAGTCGATGCCC	qPCR
15	*q 16S fw*	TCGGGAACCGTGAGACAGGT	qPCR
16	*q 16S fw*	GGACCGCTGGCAACAAAGGA	qPCR
17	*q lmo2684 fw*	CTGCAGCGCCAGGTATTTTC	qPCR
18	*q lmo2684 rev*	AGTGGTGCCAAGATGTACGG	qPCR
19	*pQE70 queT overhang fw*	AATTCACGTGGTTCATTCATACCATCCCACGTAAAAAAACTAGGAGGCATG	cloning
20	*pQE70 queT overhang rev*	CCTCCTAGTTTTTTTACGTGGGATGGTATGAATGAACCACGTG	cloning
21	*pQE70 Intergenic_lmo2684 fw*	CGGATAACAATTTCACACAGAAAGAGGGTAATTACAGC	cloning
22	*pQE70 Intergenic_lmo2684 rev*	CCTCGCCCTTGCTCAGCATGTTTTTATTCTCCTTTTTGAGGG	cloning
23	*queT i. v. Tr fw*	TGGCAAAATTGCCACACGT	PCR in vitro transcripts
24	*queT i. v. Tr rev*	CCACCCTAATCCAGAAAAGA	PCR in vitro transcripts
25	*bglA i. v. Tr fw*	GGCAAAAAGTTTCACGTCAT	PCR in vitro transcripts
26	*bglA i. v. Tr rev*	AGATACTTTAAAATGTAGAAAAGGA	PCR in vitro transcripts
27	*probe lmo2683 fw*	AATCATGTTAGTATGTTCAGCAGGT	Northern Blot
28	*probe lmo2683 rev*	GCGAAGATTTTTGCTTCAAGGC	Northern Blot
29	*probe lmo2684 fw*	CTGCAGCGCCAGGTATTTTC	Northern Blot
30	*probe lmo2684 rev*	AGTGGTGCCAAGATGTACGG	Northern Blot
31	*probe 5’ UTR lmo2684 fw*	AAAGAGGTATAGTAATAACGCCA	Northern Blot
32	*probe 5’ UTR lmo2684 rev*	TTTTTATTCTCCTTTTTGAGGGA	Northern Blot

Supporting Table S4: Mapping stats of RNA-seq results

Sample	Total reads (Mio)	Mapped (Mio)	% Mapped	% Duplicates	Properly Paired (Mio)
R1_minus_preQ1	0.95135	0.90222	94.8	0	0.900064
R2_minus_preQ1	1.281442	1.222238	95.4	0	1.218744
R3_minus_preQ1	1.081496	0.741264	68.5	0	0.739114
R1_plus_preQ1	2.559088	2.486428	97.2	0	2.480286
R2_plus_preQ1	4.136464	3.945214	95.4	0	3.931176
R3_plus_preQ1	2.915878	2.822206	96.8	0	2.813174

Supporting Table S5: Sequences used to generate the multiple sequence alignment in Fig. 4a.

RNAcentral accession #	species	Sequence (5′ to 3′)
**preQ$_1$-I type 1**		
URS0000217846_1230340	*Listeria monocytogenes*	CCACACGUGGUUCAUUCAUACCAUCCCACGUAAAAAAAACUAGGAGGAA
URS00023119CB_2126436	*Carnobacterium antarcticum*	UGUGGUUCGCAACCAUCCCACAUAAAAAAAACUAG
URS0000AB7CB8_122586	*Neisseria meningitidis*	CCGCCCGUGGUUCGAAAACCUCCCACAUUAAAAAACUAAGGAAAC
URS0000AB4454_526986	*Bacillus cereus*	AAUCACGUGGUUCGAAACCAUCCCACGUAAAAAAACUAAGGAGAU
URS0000AB3027_1590	*Lactobacillus plantarum*	CAAUACGUGGUUCGUAACCAUCCCACGUUAAAAAAACUAGGAGGAA
URS0000AB1925_71421	*Haemophilus influenzae*	CCCCCCGUAGUUCGCAAACCUCCUACAAUAAAAAACUAGGUAAAA
**preQ$_1$-I type 2**		
URS0000032866_135461	*Bacillus subtilis*	GCGGGAGAGGUUCUAGCUACACCCUCUAUAAAAAACUAAGGACGA
URS00007CF1DF_1280	*Staphylococcus aureus*	AUAUCAGAGGUUCCUAGCUGAAACCCUCUAUAAAAAACUAGACAUUG
URS0000AB9D40_428126	*Clostridium spiroforme*	AAUAAACUGGUUCGAAAGCCAAACCCAGUAUAAAAAACUAAGAAUAA
URS0000C32912_33934	*Anoxybacillus flavithermus*	GUAGGAGAGGUUCUAGAACACCCUCUAUAAAAAACUAGGGAUCG
URS0000ABC0C5_342451	*Staphylococcus saprophyticus*	UAUAAAGAGGUUCCUAGCUGAUACCCUCUAUAAAAAACUAGACACAU
URS0000C82984_866895	*Halobacillus halophilus*	ACAGGAGAGGUUCUAGCUACACCCUCUAUAAAAAACUAAGGAUGG

4. Supporting References

1. L. Flemmich and R. Micura, *Beilstein J Org Chem*, 2025, **21**, 483-489.
2. N. Ontiveros-Palacios, E. Cooke, E. P. Nawrocki, S. Triebel, M. Marz, E. Rivas, S. Griffiths-Jones, A. I. Petrov, A. Bateman and B. Sweeney, *Nucleic Acids Res*, 2025, **53**, D258-d267.
3. E. P. Nawrocki and S. R. Eddy, *Bioinformatics*, 2013, **29**, 2933-2935.
4. R. Lorenz, S. H. Bernhart, C. Höner Zu Siederdissen, H. Tafer, C. Flamm, P. F. Stadler and I. L. Hofacker, *Algorithms for molecular biology : AMB*, 2011, **6**, 26.
5. S. Janssen and R. Giegerich, *Bioinformatics*, 2015, **31**, 423-425.
6. *Nucleic Acids Res*, 2019, **47**, D1250-d1251.
7. S. Will, K. Reiche, I. L. Hofacker, P. F. Stadler and R. Backofen, *PLoS Comput Biol*, 2007, **3**, e65.
8. Y. Sato, A. Nakaya, K. Shiraishi, S. Kawashima, S. Goto and M. Kanehisa, *Genome Informatics*, 2001, **12**, 230-231.
9. S. R. Eddy, *PLoS Comput Biol*, 2011, **7**, e1002195.
10. Z. Weinberg and R. R. Breaker, *BMC Bioinformatics*, 2011, **12**, 3.
11. A. M. Waterhouse, J. B. Procter, D. M. Martin, M. Clamp and G. J. Barton, *Bioinformatics*, 2009, **25**, 1189-1191.
